# Supplementary material for: “Noncovalent Interaction”: A Chemical Misnomer That Inhibits Proper Understanding of Hydrogen Bonding, Rotation Barriers, and Other Topics
Source: Molecules. 2023 Apr 27;28(9):3776. doi: 10.3390/molecules28093776 (PMC10179974; doi:10.3390/molecules28093776)
Supplement: Supplementary file 1 [file molecules-28-03776-s001.zip › molecules-2309722-supplementary.pdf]

SUPPLEMENTARY INFORMATION: "Noncovalent Interaction": A Chemical Misnomer that Inhibits Proper Understanding of Hydrogen Bonding, Rotation Barriers, and Other Topics

F. Weinhold*

Theoretical Chemistry Institute and Department of Chemistry, University of Wisconsin-Madison, Madison, Wisconsin 53706, USA

INTRODUCTION

This SI file consists of ready-to-run *Gaussian 16* input files, each containing optimized geometrical coordinates as well as extended "title" sections summarizing vibrational frequencies, thermochemical values, and other calculated properties, followed by $NBO...$END keylist input with selected analysis keywords. Following the plan of the paper, the SI file is divided into separate "**HYDROGEN BONDING**," "**ROTATION BARRIER**," and "**STERIC REPULSION**" sections. Each section includes the input files for ordinary optimizations ("`pop=nbo7read`") followed by those for $DEL-deletion optimizations ("`pop=nbo7del`"). These files serve to re-generate all numerical values and graphical PLOT files (as displayed in the orbital images generated by the *NBOPro7@jmol* program) of the main paper.

HYDROGEN BONDING

```
%mem=2gb
%nprocshared=8
%chk=fh
#b3lyp/6-311++g** opt=calcall polar pop=nbo7read
FH, E=-100.482383390  (dE= )
nu: 4098
 Vibrational temperatures:   5895.45
          (Kelvin)
 Zero-point correction=                          0.009335 (Hartree/Particle)
 Thermal correction to Energy=                   0.011695
 Thermal correction to Enthalpy=                 0.012640
 Thermal correction to Gibbs Free Energy=       -0.007072
 Sum of electronic and zero-point Energies=          -100.473048
 Sum of electronic and thermal Energies=             -100.470688
 Sum of electronic and thermal Enthalpies=           -100.469744
 Sum of electronic and thermal Free Energies=        -100.489455
Total dipole moment    0.00  0.00 -1.98  1.98     0.00  0.00 -1.98  1.98
0 1
F
H    1   0.9222
$nbo file=fh dipole $end
--Link1--
%mem=2gb
%nprocshared=8
%chk=clh
#b3lyp/6-311++g** opt=calcall polar pop=nbo7read
ClH, E=-460.834022055  (dE= )
nu: 2934
 Vibrational temperatures:   4222.04
```

```
          (Kelvin)
 Zero-point correction=                           0.006685 (Hartree/Particle)
 Thermal correction to Energy=                    0.009046
 Thermal correction to Enthalpy=                  0.009990
 Thermal correction to Gibbs Free Energy=        -0.011204
 Sum of electronic and zero-point Energies=           -460.827337
 Sum of electronic and thermal Energies=              -460.824976
 Sum of electronic and thermal Enthalpies=            -460.824032
 Sum of electronic and thermal Free Energies=         -460.845226
Total dipole moment    0.00  0.00 -1.40  1.40     0.00  0.00 -1.40  1.40
0 1
Cl
H    1   1.2869
$nbo file=clh dipole $end
--Link1--
%mem=2gb
%nprocshared=8
%chk=brh
#b3lyp/6-311++g** opt=calcall polar pop=nbo7read
BrH, E=-2574.75317353  (dE= )
nu: 2605
 Vibrational temperatures:   3747.31
          (Kelvin)
 Zero-point correction=                           0.005934 (Hartree/Particle)
 Thermal correction to Energy=                    0.008294
 Thermal correction to Enthalpy=                  0.009238
 Thermal correction to Gibbs Free Energy=        -0.013296
 Sum of electronic and zero-point Energies=          -2574.747240
 Sum of electronic and thermal Energies=             -2574.744880
 Sum of electronic and thermal Enthalpies=           -2574.743935
 Sum of electronic and thermal Free Energies=        -2574.766470
Total dipole moment    0.00  0.00 -1.08  1.08     0.00  0.00 -1.08  1.08
0 1
Br
H    1   1.4273
$nbo file=brh dipole $end
--Link1--
%mem=2gb
%nprocshared=8
%chk=hh
#b3lyp/6-311++g** opt=calcall polar pop=nbo7read
HH, E=-1.17957156591  (dE= )
nu: 4418
 Vibrational temperatures:   6356.61
          (Kelvin)
 Zero-point correction=                           0.010065 (Hartree/Particle)
 Thermal correction to Energy=                    0.012426
 Thermal correction to Enthalpy=                  0.013370
 Thermal correction to Gibbs Free Energy=        -0.001426
 Sum of electronic and zero-point Energies=             -1.169506
 Sum of electronic and thermal Energies=                -1.167146
 Sum of electronic and thermal Enthalpies=              -1.166202
 Sum of electronic and thermal Free Energies=           -1.180997
Total dipole moment    0.00  0.00  0.00  0.00     0.00  0.00  0.00  0.00
0 1
H
H    1   0.7442
$nbo file=hh dipole $end
--Link1--
%mem=2gb
%nprocshared=8
%chk=hh_brh
#b3lyp/6-311++g** opt=calcall polar pop=nbo7read
HH...BrH, E=-2575.93273798  (dE=+0.0048)
FH, E=-100.482383390  (dE= )
ClH, E=-460.834022055  (dE= )
BrH, E=-2574.75317353  (dE= )
HH, E=-1.17957156591  (dE= )
nu: 27,31,111
 Vibrational temperatures:     39.44    44.05   159.13   185.11  3748.13
          (Kelvin)           6350.97
```

```
 Zero-point correction=                           0.016668 (Hartree/Particle)
 Thermal correction to Energy=                    0.022656
 Thermal correction to Enthalpy=                  0.023600
 Thermal correction to Gibbs Free Energy=        -0.012760
 Sum of electronic and zero-point Energies=          -2575.916070
 Sum of electronic and thermal Energies=             -2575.910082
 Sum of electronic and thermal Enthalpies=           -2575.909138
 Sum of electronic and thermal Free Energies=        -2575.945498
   1. HBr                   1.09 -0.01  0.00  1.09     1.21 -0.04  0.00  1.21
                                                 deloc: -0.12  0.03  0.00  0.12
   2. H2                    0.00  0.02  0.00  0.02     0.00  0.02  0.00  0.02
0 1
               35                 0.000000    0.217550    0.000000
                1                 1.426926    0.186963    0.000000
                1                -0.750089   -4.270970    0.000000
                1                -0.676837   -3.530234    0.000000
$nbo file=hh_brh archive nrt plot dipole $end
--Link1--
%mem=2gb
%nprocshared=8
%chk=hh_clh
#b3lyp/6-311++g** opt=calcall polar pop=nbo7read
HH...ClH, E=-462.013704938  (dE=-0.07)
FH, E=-100.482383390  (dE= )
ClH, E=-460.834022055  (dE= )
BrH, E=-2574.75317353  (dE= )
HH, E=-1.17957156591  (dE= )
nu: 40,64,146,...
 Vibrational temperatures:     57.31    91.51   210.01   234.60  4220.29
          (Kelvin)           6348.10
 Zero-point correction=                           0.017674 (Hartree/Particle)
 Thermal correction to Energy=                    0.023441
 Thermal correction to Enthalpy=                  0.024385
 Thermal correction to Gibbs Free Energy=        -0.009072
 Sum of electronic and zero-point Energies=           -461.996031
 Sum of electronic and thermal Energies=              -461.990264
 Sum of electronic and thermal Enthalpies=            -461.989320
 Sum of electronic and thermal Free Energies=         -462.022777
   1. HCl                   1.41 -0.12  0.00  1.41     1.57 -0.18  0.00  1.58
                                                 deloc: -0.17  0.06  0.00  0.18
   2. H2                    0.00  0.03  0.00  0.03     0.00  0.03  0.00  0.03
0 1
               17                 0.000000    0.380003   -0.000000
                1                 1.280477    0.250120   -0.000000
                1                -0.658932   -3.726854    0.000000
                1                -0.621544   -2.983324    0.000000
$nbo file=hh_clh archive nrt plot dipole $end
--Link1--
%mem=2gb
%nprocshared=8
%chk=hh_fh
#b3lyp/6-311++g** opt=calcall polar pop=nbo7read
HH...FH, E=-101.662377949  (dE=-0.27)
FH, E=-100.482383390  (dE= )
ClH, E=-460.834022055  (dE= )
BrH, E=-2574.75317353  (dE= )
HH, E=-1.17957156591  (dE= )
nu: 79,116,227,...
 Vibrational temperatures:    114.15   167.55   326.67   340.04  5890.74
          (Kelvin)           6338.88
 Zero-point correction=                           0.020866 (Hartree/Particle)
 Thermal correction to Energy=                    0.026203
 Thermal correction to Enthalpy=                  0.027147
 Thermal correction to Gibbs Free Energy=        -0.002526
 Sum of electronic and zero-point Energies=           -101.641512
 Sum of electronic and thermal Energies=              -101.636175
 Sum of electronic and thermal Enthalpies=            -101.635231
 Sum of electronic and thermal Free Energies=         -101.664904
   1. HF                    1.61  1.18  0.00  1.99     1.79  1.30  0.00  2.21
                                                 deloc: -0.18 -0.12  0.00  0.22
   2. H2                    0.00  0.08  0.00  0.08     0.01  0.08  0.00  0.09
```

```
0 1
               9                   0.000000    0.451609    0.000000
               1                   0.746640    0.993348    0.000000
               1                  -0.399769   -2.900430   -0.000000
               1                  -0.346871   -2.157402   -0.000000
$nbo file=hh_fh archive nrt plot dipole $end
--Link1--
%mem=2gb
%nprocshared=8
%chk=brh_brh
#b3lyp/6-311++g** opt=calcall polar pop=nbo7read
BrH...BrH, E= -5149.50797736  (dE=-1.02)
FH, E=-100.482383390  (dE= )
ClH, E=-460.834022055  (dE= )
BrH, E=-2574.75317353  (dE= )
HH, E=-1.17957156591  (dE= )
nu: 37,96,173,...
 Vibrational temperatures:     53.85   138.23   248.35   372.90  3659.65
          (Kelvin)           3741.29
 Zero-point correction=                           0.013007 (Hartree/Particle)
 Thermal correction to Energy=                    0.018521
 Thermal correction to Enthalpy=                  0.019466
 Thermal correction to Gibbs Free Energy=        -0.018302
 Sum of electronic and zero-point Energies=          -5149.494971
 Sum of electronic and thermal Energies=             -5149.489456
 Sum of electronic and thermal Enthalpies=           -5149.488512
 Sum of electronic and thermal Free Energies=        -5149.526279
   1. HBr                   -1.13 -0.40  0.00  1.20    -1.24  0.00  0.00  1.24
                                                    deloc:  0.11 -0.40  0.00  0.42
   2. HBr                    0.13 -1.20  0.00  1.20     0.13 -1.34  0.00  1.35
0 1
              35                   0.018027   -2.102222    0.000000
               1                  -1.408607   -2.168100    0.000000
              35                   0.018027    2.143678    0.000000
               1                   0.146735    0.717134    0.000000
$nbo file=brh_brh archive nrt plot dipole $end
--Link1--
%mem=2gb
%nprocshared=8
%chk=brh_clh
#b3lyp/6-311++g** opt=calcall polar pop=nbo7read
BrH...ClH, E=-3035.58908266  (dE=-1.18)
FH, E=-100.482383390  (dE= )
ClH, E=-460.834022055  (dE= )
BrH, E=-2574.75317353  (dE= )
HH, E=-1.17957156591  (dE= )
nu: 48,105,167...
 Vibrational temperatures:     68.96   150.84   239.97   391.88  3685.98
          (Kelvin)           4209.92
 Zero-point correction=                           0.013851 (Hartree/Particle)
 Thermal correction to Energy=                    0.019319
 Thermal correction to Enthalpy=                  0.020263
 Thermal correction to Gibbs Free Energy=        -0.016123
 Sum of electronic and zero-point Energies=          -3035.575232
 Sum of electronic and thermal Energies=             -3035.569764
 Sum of electronic and thermal Enthalpies=           -3035.568820
 Sum of electronic and thermal Free Energies=        -3035.605205
   1. HCl                   -1.44  0.40  0.00  1.50    -1.60  0.13  0.00  1.61
                                                    deloc:  0.16  0.27  0.00  0.31
   2. HBr                    0.17  1.19  0.00  1.20     0.18  1.34  0.00  1.35
                                                    deloc: -0.01 -0.16  0.00  0.16
0 1
              17                   0.020399    2.724414   -0.000000
               1                  -1.259279    2.870995    0.000000
              35                   0.020399   -1.405720    0.000000
               1                   0.198506    0.014166   -0.000000
$nbo file=brh_clh archive nrt plot dipole $end
--Link1--
%mem=2gb
%nprocshared=8
%chk=brh_fh
```

```
#b3lyp/6-311++g** opt=calcall polar pop=nbo7read
BrH...FH, E=-2675.23940475  (dE=-2.41)
FH, E=-100.482383390  (dE= )
ClH, E=-460.834022055  (dE= )
BrH, E=-2574.75317353  (dE= )
HH, E=-1.17957156591  (dE= )
nu: 87,135,235,...
 Vibrational temperatures:    125.01   194.36   338.82   441.36  3701.25
          (Kelvin)           5860.50
 Zero-point correction=                           0.016881 (Hartree/Particle)
 Thermal correction to Energy=                    0.022062
 Thermal correction to Enthalpy=                  0.023007
 Thermal correction to Gibbs Free Energy=        -0.010771
 Sum of electronic and zero-point Energies=          -2675.222524
 Sum of electronic and thermal Energies=             -2675.217342
 Sum of electronic and thermal Enthalpies=           -2675.216398
 Sum of electronic and thermal Free Energies=        -2675.250175
   1. HF                    -1.64  1.34  0.00  2.12    -1.81  1.30  0.00  2.23
                                                 deloc:  0.17  0.04  0.00  0.17
   2. HBr                    0.13  1.34  0.00  1.34     0.14  1.50  0.00  1.51
                                                 deloc:  0.00 -0.16  0.00  0.16
0 1
                9                  0.013843    2.729208   -0.000000
                1                 -0.732269    3.274315    0.000000
               35                  0.013843   -0.812897   -0.000000
                1                  0.123170    0.614227   -0.000000
$nbo file=brh_fh archive nrt plot dipole $end
--Link1--
%mem=2gb
%nprocshared=8
%chk=clh_brh
#b3lyp/6-311++g** opt=calcall polar pop=nbo7read
ClH...BrH, E=-3035.58948228  (dE=-1.44)
FH, E=-100.482383390  (dE= )
ClH, E=-460.834022055  (dE= )
BrH, E=-2574.75317353  (dE= )
HH, E=-1.17957156591  (dE= )
nu: 55,108,207,...
 Vibrational temperatures:     79.77   154.75   297.36   425.11  3739.48
          (Kelvin)           4097.27
 Vibrational temperatures:     79.77   154.75   297.36   425.11  3739.48
          (Kelvin)           4097.27
 Zero-point correction=                           0.013924 (Hartree/Particle)
 Thermal correction to Energy=                    0.019277
 Thermal correction to Enthalpy=                  0.020221
 Thermal correction to Gibbs Free Energy=        -0.015784
 Sum of electronic and zero-point Energies=          -3035.575558
 Sum of electronic and thermal Energies=             -3035.570206
 Sum of electronic and thermal Enthalpies=           -3035.569261
 Sum of electronic and thermal Free Energies=        -3035.605266
   1. HBr                    0.47  1.14  0.00  1.24     0.04  1.25  0.00  1.25
                                                 deloc:  0.43 -0.11  0.00  0.45
   2. HCl                    1.50 -0.16  0.00  1.51     1.71 -0.17  0.00  1.71
                                                 deloc: -0.20  0.01  0.00  0.20
0 1
               35                 -1.309859   -0.039403    0.000002
                1                 -1.396827    1.386314   -0.000016
               17                  2.696059    0.006404    0.000008
                1                  1.408902   -0.116073   -0.000211
$nbo file=clh_brh archive nrt plot dipole $end
--Link1--
%mem=2gb
%nprocshared=8
%chk=clh_clh
#b3lyp/6-311++g** opt=calcall polar pop=nbo7read
ClH...ClH, E=-921.670558460  (dE=-1.58)
FH, E=-100.482383390  (dE= )
ClH, E=-460.834022055  (dE= )
BrH, E=-2574.75317353  (dE= )
HH, E=-1.17957156591  (dE= )
nu: 65,113,199,...
```

```
 Vibrational temperatures:     93.22   163.27   286.10   445.42  4129.22
          (Kelvin)           4208.27
 Zero-point correction=                           0.014766 (Hartree/Particle)
 Thermal correction to Energy=                    0.020083
 Thermal correction to Enthalpy=                  0.021027
 Thermal correction to Gibbs Free Energy=        -0.013646
 Sum of electronic and zero-point Energies=           -921.655792
 Sum of electronic and thermal Energies=              -921.650476
 Sum of electronic and thermal Enthalpies=            -921.649531
 Sum of electronic and thermal Free Energies=         -921.684204
   1. HCl                    -0.45  1.45  0.00  1.52    -0.15  1.61  0.00  1.62
                                                 deloc: -0.30 -0.16  0.00  0.34
   2. HCl                    -1.49 -0.26  0.00  1.51    -1.70 -0.28  0.00  1.72
                                                 deloc:  0.21  0.02  0.00  0.21
0 1
              17                  -1.904641   -0.070217   -0.000015
               1                  -2.057085    1.208964    0.000074
              17                   1.983989    0.010337   -0.000021
               1                   0.708167   -0.190990    0.000546
$nbo file=clh_clh archive nrt plot dipole $end
--Link1--
%mem=2gb
%nprocshared=8
%chk=clh_fh
#b3lyp/6-311++g** opt=calcall polar pop=nbo7read
ClH...FH, E=-561.321312583  (dE=-3.08)
FH, E=-100.482383390  (dE= )
ClH, E=-460.834022055  (dE= )
BrH, E=-2574.75317353  (dE= )
HH, E=-1.17957156591  (dE= )
nu: 112,161,292,....
 Vibrational temperatures:    160.70   232.10   419.69   523.81  4141.07
          (Kelvin)           5854.40
 Zero-point correction=                           0.017943 (Hartree/Particle)
 Thermal correction to Energy=                    0.022888
 Thermal correction to Enthalpy=                  0.023833
 Thermal correction to Gibbs Free Energy=        -0.008107
 Sum of electronic and zero-point Energies=           -561.303370
 Sum of electronic and thermal Energies=              -561.298424
 Sum of electronic and thermal Enthalpies=            -561.297480
 Sum of electronic and thermal Free Energies=         -561.329420
   1. HF                     -1.69  1.30  0.00  2.14    -1.86  1.23  0.00  2.23
                                                 deloc:  0.17  0.07  0.00  0.18
   2. HCl                     0.17  1.63  0.00  1.64     0.18  1.85  0.00  1.85
                                                 deloc: -0.02 -0.22  0.00  0.22
0 1
               9                   0.023339    2.067101   -0.000000
               1                  -0.742198    2.585194   -0.000000
              17                   0.023339   -1.248692    0.000000
               1                   0.135379    0.038657   -0.000000
$nbo file=clh_fh archive nrt plot dipole $end
--Link1--
%mem=2gb
%nprocshared=8
%chk=fh_brh
#b3lyp/6-311++g** opt=calcall polar pop=nbo7read
FH...BrH, E=-2675.23977260  (dE=-2.65)
FH, E=-100.482383390  (dE= )
ClH, E=-460.834022055  (dE= )
BrH, E=-2574.75317353  (dE= )
HH, E=-1.17957156591  (dE= )
nu: 96,145,337,...
 Vibrational temperatures:    138.07   209.26   485.16   642.11  3734.92
          (Kelvin)           5663.42
 Zero-point correction=                           0.017216 (Hartree/Particle)
 Thermal correction to Energy=                    0.022085
 Thermal correction to Enthalpy=                  0.023029
 Thermal correction to Gibbs Free Energy=        -0.010544
 Sum of electronic and zero-point Energies=          -2675.222556
 Sum of electronic and thermal Energies=             -2675.217687
 Sum of electronic and thermal Enthalpies=           -2675.216743
```

```
 Sum of electronic and thermal Free Energies=        -2675.250317
   1. HBr                   -0.61  1.18  0.00  1.33    -0.09  1.28  0.00  1.29
                                               deloc: -0.52 -0.10  0.00  0.53
   2. HF                    -2.06 -0.26  0.00  2.08    -2.29 -0.28  0.00  2.31
                                               deloc:  0.23  0.02  0.00  0.23
0 1
               35                 -0.718359   -0.039414   -0.000002
                1                 -0.763678    1.388916    0.000015
                9                  2.682823    0.010399   -0.000017
                1                  1.760836   -0.103009    0.000212
$nbo file=fh_brh archive nrt plot dipole $end
--Link1--
%mem=2gb
%nprocshared=8
%chk=fh_clh
#b3lyp/6-311++g** opt=calcall polar pop=nbo7read
FH...ClH, E=-561.320823712  (dE=-2.77)
FH, E=-100.482383390  (dE= )
ClH, E=-460.834022055  (dE= )
BrH, E=-2574.75317353  (dE= )
HH, E=-1.17957156591  (dE= )
nu: 104,155,344...
 Vibrational temperatures:    150.11   223.64   494.92   670.92  4199.60
          (Kelvin)           5695.20
 Zero-point correction=                           0.018105 (Hartree/Particle)
 Thermal correction to Energy=                    0.022916
 Thermal correction to Enthalpy=                  0.023861
 Thermal correction to Gibbs Free Energy=        -0.008327
 Sum of electronic and zero-point Energies=           -561.302718
 Sum of electronic and thermal Energies=              -561.297907
 Sum of electronic and thermal Enthalpies=            -561.296963
 Sum of electronic and thermal Free Energies=         -561.329151
   1. HCl                   -0.55  1.49  0.00  1.59    -0.14  1.65  0.00  1.65
                                               deloc: -0.41 -0.15  0.00  0.43
   2. HF                    -2.04 -0.38  0.00  2.08    -2.27 -0.41  0.00  2.31
                                               deloc:  0.23  0.04  0.00  0.23
0 1
               17                 -1.128882   -0.071457   -0.000003
                1                 -1.248200    1.212058    0.000013
                9                  2.135246    0.016820   -0.000009
                1                  1.221986   -0.148675    0.000114
$nbo file=fh_clh archive nrt plot dipole $end
--Link1--
%mem=2gb
%nprocshared=8
%chk=fh_fh
#b3lyp/6-311++g** opt=calcall polar pop=nbo7read
FH...FH, E=-200.972811239  (dE=-5.05)
FH, E=-100.482383390  (dE= )
ClH, E=-460.834022055  (dE= )
BrH, E=-2574.75317353  (dE= )
HH, E=-1.17957156591  (dE= )
nu: 161,220,456...
 Vibrational temperatures:    232.18   317.65   655.94   829.39  5695.68
          (Kelvin)           5838.65
 Zero-point correction=                           0.021486 (Hartree/Particle)
 Thermal correction to Energy=                    0.025903
 Thermal correction to Enthalpy=                  0.026848
 Thermal correction to Gibbs Free Energy=        -0.002861
 Sum of electronic and zero-point Energies=           -200.951325
 Sum of electronic and thermal Energies=              -200.946908
 Sum of electronic and thermal Enthalpies=            -200.945964
 Sum of electronic and thermal Free Energies=         -200.975672
   1. HF                    -1.86 -1.13  0.00  2.18    -2.04 -0.96  0.00  2.25
                                               deloc:  0.18 -0.17  0.00  0.25
   2. HF                     0.31 -2.13  0.00  2.15     0.34 -2.38  0.00  2.40
                                               deloc: -0.03  0.25  0.00  0.25
0 1
                9                  0.034957   -1.306771   -0.000000
                1                 -0.793920   -1.718253   -0.000000
                9                  0.034957    1.439904    0.000000
```

```
                1                   0.164698    0.520062    0.000000
$nbo file=fh_fh archive nrt plot dipole $end
--Link1--
--Link1--
%mem=2gb
%nprocshared=8
%chk=xh_yh
#b3lyp/6-311++g** opt=calcall polar pop=nbo7read
XH...YH, E=  (dE= )
0 1
Y
H    1   1.
X    1   3.   2   110.
H    3   1.   2    30.    1    0.
$nbo file=xh_yh archive nrt plot dipole $end
--Link1--
--Link1--
%mem=2gb
%nprocshared=8
%chk=xh_yh
#b3lyp/6-311++g** nosymm opt=(maxcycles=100) pop=nbo7del
HH...BrH, E($DEL)=-2575.932652176 (dE=0.06)
0 1
 Br
 H   1    r1
 x   1     1.      2     90.
 H   1    r2       2     a1       3     10.
 x   4     1.      1     90.      3     180.
 H   4    r3       5     a2       1     tw
r1   1.4270
r2   4.3650
r3   0.7443
a1   186.13
a2   -45.89
tw   -101.31
$nbo print=0 $end
$del
  delete 2 orbitals 20 21
$end
--Link1--
--Link1--
%mem=2gb
%nprocshared=8
%chk=xh_yh
#b3lyp/6-311++g** nosymm opt=(maxcycles=100) pop=nbo7del
FH...FH, E($DEL)=  (dE= )
0 1
 F
 H   1    r1
 x   1     1.      2     90.
 F   1    r2       2     a1       3     10.
 x   4     1.      1     90.      3     180.
 H   4    r3       5     a2       1     tw
r1   1.
r2   3.
r3   1.
a1   120.
a2   90.
tw   20.
$nbo print=0 $end
$del
  delete 2 orbitals 11 12
$end
--Link1--
--Link1--
%mem=2gb
%nprocshared=8
%chk=xh_yh
#b3lyp/6-311++g** nosymm opt=(maxcycles=100) pop=nbo7del
FH...FH, E($DEL)=-200.969569707  (dE=-3.01)
0 1
```

```
 F
 H   1    r1
 x   1     1.      2     90.
 F   1    r2       2     a1       3     10.
 x   4     1.      1     90.      3     180.
 H   4    r3       5     a2       1     tw
r1   0.9239
r2   3.1797
r3   0.9227
a1   129.70
a2   76.36
tw   -3.84
$nbo print=0 $end
$del
  delete 2 orbitals 11 12
$end
--Link1--
--Link1--
%mem=2gb
%nprocshared=8
%chk=xh_yh
#b3lyp/6-311++g** nosymm opt=(maxcycles=100) pop=nbo7del
FH...ClH, E($DEL)=-561.319081897  (dE=)
               17           0        0.000000    0.000000    0.000000
                1           0        0.000000    0.000000    1.286382
                9           0        1.950572   -0.343938    2.935405
                1           0        2.791390   -0.334628    2.553954
0 1
 Cl
 H   1    r1
 x   1     1.      2     90.
 F   1    r2       2     a1       3     10.
 x   4     1.      1     90.      3     180.
 H   4    r3       5     a2       1     tw
r1   1.2864
r2   3.5411
r3   0.9233
a1   34.01
a2   189.36
tw   13.53
$nbo print=0 $end
$del
  delete 2 orbitals 15 16
$end
--Link1--
--Link1--
%mem=2gb
%nprocshared=8
%chk=xh_yh
#b3lyp/6-311++g** nosymm opt=(maxcycles=100) pop=nbo7del
FH...BrH, E($DEL)=-2675.237584089  (dE=-1.27)
              35                   0.000000    0.000000    0.000000
               1                   0.000000    0.000000    1.426300
               9                   2.444453   -0.431023    2.691414
               1                   2.990902   -0.343013    1.952644
0 1
 Br
 H   1    r1
 x   1     1.      2     90.
 F   1    r2       2     a1       3     10.
 x   4     1.      1     90.      3     180.
 H   4    r3       5     a2       1     tw
r1   1.4263
r2   3.316613
r3   0.9231
a1   42.68
a2   168.06
tw   -9.01
$nbo print=0 $end
$del
  delete 2 orbitals 24 25
```

```
$end
--Link1--
--Link1--
%mem=2gb
%nprocshared=8
%chk=xh_yh
#b3lyp/6-311++g** nosymm opt=(maxcycles=100) pop=nbo7del
ClH...FH, E($DEL)=-561.319088228  (dE=-1.68 )
0 1
 F
 H   1    r1
 x   1     1.      2     90.
 Cl  1    r2       2     a1       3     10.
 x   4     1.      1     90.      3     180.
 H   4    r3       5     a2       1     tw
r1   0.9232
r2   3.6872
r3   1.2861
a1   116.69
a2   67.54
tw   -9.16
$nbo print=0 $end
$del
  delete 2 orbitals 15 16
$end
--Link1--
--Link1--
%mem=2gb
%nprocshared=8
%chk=xh_yh
#b3lyp/6-311++g** nosymm opt=(maxcycles=100) pop=nbo7del
ClH...ClH, E($DEL)=-921.669650599  (dE=-1.01)
0 1
 Cl
 H   1    r1
 x   1     1.      2     90.
 Cl  1    r2       2     a1       3     10.
 x   4     1.      1     90.      3     180.
 H   4    r3       5     a2       1     tw
r1   1.2874
r2   4.0333
r3   1.2871
a1   54.84
a2   143.60
tw   -15.53
$nbo print=0 $end
$del
  delete 2 orbitals 19 20
$end
--Link1--
--Link1--
%mem=2gb
%nprocshared=8
%chk=xh_yh
#b3lyp/6-311++g** nosymm opt=(maxcycles=100) pop=nbo7del
ClH...BrH, E($DEL)=-3035.587950624  (dE=-0.47)
0 1
 Br
 H   1    r1
 x   1     1.      2     90.
 Cl  1    r2       2     a1       3     10.
 x   4     1.      1     90.      3     180.
 H   4    r3       5     a2       1     tw
r1   1.4272
r2   5.0997
r3   1.2870
a1   101.36
a2   82.63
tw   0.01
$nbo print=0 $end
$del
```

```
  delete 2 orbitals 28 29
$end
--Link1--
--Link1--
%mem=2gb
%nprocshared=8
%chk=xh_yh
#b3lyp/6-311++g** nosymm opt=(maxcycles=100) pop=nbo7del
BrH...FH, E($DEL)=-2675.237558498  (dE=-1.26)
0 1
 F
 H   1    r1
 x   1     1.      2     90.
 Br  1    r2       2     a1       3     10.
 x   4     1.      1     90.      3     180.
 H   4    r3       5     a2       1     tw
r1   0.9230
r2   3.9294
r3   1.4261
a1   117.52
a2   67.26
tw   -9.03
$nbo print=0 $end
$del
  delete 2 orbitals 24 25
$end
--Link1--
--Link1--
%mem=2gb
%nprocshared=8
%chk=xh_yh
#b3lyp/6-311++g** nosymm opt=(maxcycles=100) pop=nbo7del
BrH...ClH, E($DEL)=-3035.588046795 (dE=-0.53)
0 1
 Cl
 H   1    r1
 x   1     1.      2     90.
 Br  1    r2       2     a1       3     10.
 x   4     1.      1     90.      3     180.
 H   4    r3       5     a2       1     tw
r1   1.2870
r2   4.8190
r3   1.4265
a1   96.41
a2   77.05
tw   -13.65
$nbo print=0 $end
$del
  delete 2 orbitals 28 29
$end
--Link1--
--Link1--
%mem=2gb
%nprocshared=8
%chk=xh_yh
#b3lyp/6-311++g** nosymm opt=(maxcycles=100) pop=nbo7del
BrH...BrH, E($DEL)=-5149.506516181* (dE=-0.11)
0 1
 Br
 H   1    r1
 x   1     1.      2     90.
 Br  1    r2       2     a1       3     10.
 x   4     1.      1     90.      3     180.
 H   4    r3       5     a2       1     tw
r1   1.4261
r2   4.6019
r3   1.4292
a1   221.98
a2   42.66
tw   2.67
$nbo print=0 $end
```

```
$del
  delete 2 orbitals 37 38
$end
--Link1--
--Link1--
%mem=2gb
%nprocshared=8
%chk=xh_yh
#b3lyp/6-311++g** nosymm opt=(maxcycles=100) pop=nbo7del
HH...FH, E($DEL)=-101.662438626 (dE=-0.30)
0 1
 F
 H   1    r1
 x   1     1.      2     90.
 H   1    r2       2     a1       3     10.
 x   4     1.      1     90.      3     180.
 H   4    r3       5     a2       1     tw
r1   0.9222
r2   3.4401
r3   0.7451
a1   5.65
a2   352.25
tw   -213.18
$nbo print=0 $end
$del
  delete 2 orbitals 7 8
$end
--Link1--
--Link1--
%mem=2gb
%nprocshared=8
%chk=xh_yh
#b3lyp/6-311++g** nosymm opt=(maxcycles=100) pop=nbo7del
HH...ClH, E($DEL)=-462.013682447 (dE=-0.06)
0 1
 Cl
 H   1    r1
 x   1     1.      2     90.
 H   1    r2       2     a1       3     10.
 x   4     1.      1     90.      3     180.
 H   4    r3       5     a2       1     tw
r1   1.2876
r2   5.3140
r3   0.7429
a1   12.66
a2   42.14
tw   103.34
$nbo print=0 $end
$del
  delete 2 orbitals 11 12
$end
--Link1--
--Link1—
```

ROTATION BARRIERS

```
----
HOOH
----
full: -151.6021697 @ 121.12
 DEL: -151.5777827 @ 130.44
%-Del-E = 0.016%
------
CH3CH3
------
full: -79.8565753 @ 60.12
 DEL: -79.8179280 @ 63.26
%-Del-E = 0.048%
------
CH3NH2
------
```

```
full: -95.8938910
 DEL: -95.8466542
%-Del-E = 0.049%
------
HCONH2
------
full: -169.954848506
 DEL: -169.769538009
%-Del-E = 0.684%
---------------------------------------------------------------------------
%mem=2gb
%nprocshared=8
%chk=rot_barr
#b3lyp/6-311++g** pop=nbo7del nosymm opt=z-matrix
HCONH2 (tau=0), E(DEL)=-169.767055457 (1.56)
0 1
H
C   1  r2
O   2  r3   1   a3
N   2  r4   1   a4   3  d4
H   4  r5   2   a5   3  tau
H   4  r6   2   a6   3  d6
r2   1.0500
r3   1.2982
a3   116.52
r4   1.5477
a4   130.02
d4   178.47
r5   1.0094
a5   105.48
r6   1.0096
a6   107.92
d6   246.08
tau  0.
$nbo print=0 $end
$DEL
 Lewis
$END
--Link1--
%mem=2gb
%nprocshared=8
%chk=rot_barr
#b3lyp/6-311++g** pop=nbo7del nosymm opt=z-matrix
HCONH2 (tau=10), E(DEL)=-169.767655109 (1.18)
0 1
H
C   1  r2
O   2  r3   1   a3
N   2  r4   1   a4   3  d4
H   4  r5   2   a5   3  tau
H   4  r6   2   a6   3  d6
r2   1.0496
r3   1.2994
a3   116.64
r4   1.5469
a4   129.72
d4   177.93
r5   1.0105
a5   105.37
r6   1.0108
a6   107.67
d6   256.39
tau  10.
$nbo print=0 $end
$DEL
 Lewis
$END
--Link1--
%mem=2gb
%nprocshared=8
%chk=rot_barr
```

```
#b3lyp/6-311++g** pop=nbo7del nosymm opt=z-matrix
HCONH2 (tau=20), E(DEL)=-169.768249692 (0.81)
0 1
H
C   1  r2
O   2  r3   1   a3
N   2  r4   1   a4   3  d4
H   4  r5   2   a5   3  tau
H   4  r6   2   a6   3  d6
r2   1.0490
r3   1.3007
a3   116.96
r4   1.5452
a4   129.18
d4   177.62
r5   1.0112
a5   105.37
r6   1.0116
a6   107.42
d6   266.61
tau  20.
$nbo print=0 $end
$DEL
 Lewis
$END
--Link1--
%mem=2gb
%nprocshared=8
%chk=rot_barr
#b3lyp/6-311++g** pop=nbo7del nosymm opt=z-matrix
HCONH2 (tau=30), E(DEL)= -169.768798741 (0.46)
0 1
H
C   1  r2
O   2  r3   1   a3
N   2  r4   1   a4   3  d4
H   4  r5   2   a5   3  tau
H   4  r6   2   a6   3  d6
r2   1.0486
r3   1.3019
a3   117.17
r4   1.5437
a4   128.80
d4   177.72
r5   1.0122
a5   105.40
r6   1.0126
a6   107.02
d6   277.02
tau  30.
$nbo print=0 $end
$DEL
 Lewis
$END
--Link1--
%mem=2gb
%nprocshared=8
%chk=rot_barr
#b3lyp/6-311++g** pop=nbo7del nosymm opt=z-matrix
HCONH2 (tau=40), E(DEL)=-169.769234708 (0.19)
0 1
H
C   1  r2
O   2  r3   1   a3
N   2  r4   1   a4   3  d4
H   4  r5   2   a5   3  tau
H   4  r6   2   a6   3  d6
r2   1.0482
r3   1.3029
a3   117.34
r4   1.5421
```

```
a4   128.50
d4   178.25
r5   1.0129
a5   105.58
r6   1.0131
a6   106.65
d6   287.20
tau  40.
$nbo print=0 $end
$DEL
 Lewis
$END
--Link1--
%mem=2gb
%nprocshared=8
%chk=rot_barr
#b3lyp/6-311++g** pop=nbo7del nosymm opt=z-matrix
HCONH2 (tau=50), E(DEL)=-169.769489864 (0.03)
0 1
H
C   1  r2
O   2  r3   1   a3
N   2  r4   1   a4   3  d4
H   4  r5   2   a5   3  tau
H   4  r6   2   a6   3  d6
r2   1.0479
r3   1.3036
a3   117.49
r4   1.5415
a4   128.38
d4   179.32
r5   1.0130
a5   105.84
r6   1.0131
a6   106.28
d6   297.14
tau  50.
$nbo print=0 $end
$DEL
 Lewis
$END
--Link1--
%mem=2gb
%nprocshared=8
%chk=rot_barr
#b3lyp/6-311++g** pop=nbo7del nosymm opt=z-matrix
HCONH2 (tau=60), E(DEL)=-169.769525017 (0.01)
0 1
H
C   1  r2
O   2  r3   1   a3
N   2  r4   1   a4   3  d4
H   4  r5   2   a5   3  tau
H   4  r6   2   a6   3  d6
r2   1.0479
r3   1.3037
a3   117.53
r4   1.5403
a4   128.22
d4   180.46
r5   1.0130
a5   106.32
r6   1.0130
a6   106.08
d6   306.78
tau  60.
$nbo print=0 $end
$DEL
 Lewis
$END
--Link1--
```

```
%mem=2gb
%nprocshared=8
%chk=rot_barr
#b3lyp/6-311++g** pop=nbo7del nosymm opt=z-matrix
HCONH2 (tau=70), E(DEL)=-169.768978994 (0.35)
0 1
H
C   1  r2
O   2  r3   1   a3
N   2  r4   1   a4   3  d4
H   4  r5   2   a5   3  tau
H   4  r6   2   a6   3  d6
r2   1.0484
r3   1.3022
a3   117.29
r4   1.5419
a4   128.54
d4   182.22
r5   1.0117
a5   107.32
r6   1.0115
a6   105.88
d6   325.47
tau  70.
$nbo print=0 $end
$DEL
 Lewis
$END
--Link1--
%mem=2gb
%nprocshared=8
%chk=rot_barr
#b3lyp/6-311++g** pop=nbo7del nosymm opt=z-matrix
HCONH2 (tau=80), E(DEL)=-169.768978994 (0.35)
0 1
H
C   1  r2
O   2  r3   1   a3
N   2  r4   1   a4   3  d4
H   4  r5   2   a5   3  tau
H   4  r6   2   a6   3  d6
r2   1.0484
r3   1.3022
a3   117.29
r4   1.5419
a4   128.54
d4   182.22
r5   1.0117
a5   107.32
r6   1.0115
a6   105.88
d6   325.47
tau  80.
$nbo print=0 $end
$DEL
 Lewis
$END
--Link1--
%mem=2gb
%nprocshared=8
%chk=rot_barr
#b3lyp/6-311++g** pop=nbo7del nosymm opt=z-matrix
HCONH2 (tau=90), E(DEL)=-169.768490053 (0.66)
0 1
H
C   1  r2
O   2  r3   1   a3
N   2  r4   1   a4   3  d4
H   4  r5   2   a5   3  tau
H   4  r6   2   a6   3  d6
r2   1.0488
```

```
r3   1.3011
a3   117.08
r4   1.5431
a4   128.86
d4   182.46
r5   1.0106
a5   107.80
r6   1.0104
a6   105.91
d6   334.74
tau  90.
$nbo print=0 $end
$DEL
 Lewis
$END
--Link1--
%mem=2gb
%nprocshared=8
%chk=rot_barr
#b3lyp/6-311++g** pop=nbo7del nosymm opt=z-matrix
HCONH2 (tau=100), E(DEL)=-169.767931662 (1.01)
0 1
H
C   1  r2
O   2  r3   1   a3
N   2  r4   1   a4   3  d4
H   4  r5   2   a5   3  tau
H   4  r6   2   a6   3  d6
r2   1.0493
r3   1.2998
a3   116.88
r4   1.5445
a4   129.24
d4   182.44
r5   1.0097
a5   108.14
r6   1.0096
a6   105.94
d6   344.33
tau  100.
$nbo print=0 $end
$DEL
 Lewis
$END
--Link1--
%mem=2gb
%nprocshared=8
%chk=rot_barr
#b3lyp/6-311++g** pop=nbo7del nosymm opt=z-matrix
HCONH2 (tau=110), E(DEL)=-169.767349406 (1.37)
0 1
H
C   1  r2
O   2  r3   1   a3
N   2  r4   1   a4   3  d4
H   4  r5   2   a5   3  tau
H   4  r6   2   a6   3  d6
r2   1.0498
r3   1.2986
a3   116.69
r4   1.5453
a4   129.68
d4   182.03
r5   1.0085
a5   108.48
r6   1.0085
a6   106.04
d6   353.82
tau  110.
$nbo print=0 $end
$DEL
```

```
 Lewis
$END
--Link1--
%mem=2gb
%nprocshared=8
%chk=rot_barr
#b3lyp/6-311++g** pop=nbo7del nosymm opt=z-matrix
HCONH2 (tau=120), E(DEL)=-169.766761745 (1.74)
0 1
H
C   1  r2
O   2  r3   1   a3
N   2  r4   1   a4   3  d4
H   4  r5   2   a5   3  tau
H   4  r6   2   a6   3  d6
r2   1.0503
r3   1.2975
a3   116.50
r4   1.5461
a4   130.16
d4   181.34
r5   1.0076
a5   108.65
r6   1.0078
a6   106.04
d6   363.70
tau  120.
$nbo print=0 $end
$DEL
 Lewis
$END
--Link1--
%mem=2gb
%nprocshared=8
%chk=rot_barr
#b3lyp/6-311++g** pop=nbo7del nosymm opt=z-matrix
HCONH2 (tau=130), E(DEL)=-169.761210536 (5.22)
0 1
H
C   1  r2
O   2  r3   1   a3
N   2  r4   1   a4   3  d4
H   4  r5   2   a5   3  tau
H   4  r6   2   a6   3  d6
r2   1.0481
r3   1.3020
a3   117.19
r4   1.5345
a4   125.52
d4   181.91
r5   1.0080
a5   109.77
r6   1.0081
a6   109.40
d6   250.60
tau  130.
$nbo print=0 $end
$DEL
 Lewis
$END
--Link1--
%mem=2gb
%nprocshared=8
%chk=rot_barr
#b3lyp/6-311++g** pop=nbo7del nosymm opt=z-matrix
HCONH2 (tau=140), E(DEL)=-169.761016586 (5.35)
0 1
H
C   1  r2
O   2  r3   1   a3
N   2  r4   1   a4   3  d4
```

```
H   4  r5   2   a5   3  tau
H   4  r6   2   a6   3  d6
r2   1.0484
r3   1.3015
a3   117.09
r4   1.5358
a4   125.77
d4   183.53
r5   1.0079
a5   109.80
r6   1.0080
a6   109.14
d6   260.26
tau  140.
$nbo print=0 $end
$DEL
 Lewis
$END
--Link1--
%mem=2gb
%nprocshared=8
%chk=rot_barr
#b3lyp/6-311++g** pop=nbo7del nosymm opt=z-matrix
HCONH2 (tau=150), E(DEL)=-169.760840721 (5.46)
0 1
H
C   1  r2
O   2  r3   1   a3
N   2  r4   1   a4   3  d4
H   4  r5   2   a5   3  tau
H   4  r6   2   a6   3  d6
r2   1.0488
r3   1.3008
a3   116.92
r4   1.5374
a4   126.26
d4   184.33
r5   1.0074
a5   109.86
r6   1.0075
a6   109.00
d6   270.28
tau  150.
$nbo print=0 $end
$DEL
 Lewis
$END
--Link1--
%mem=2gb
%nprocshared=8
%chk=rot_barr
#b3lyp/6-311++g** pop=nbo7del nosymm opt=z-matrix
HCONH2 (tau=160), E(DEL)=-169.760844945 (5.46)
0 1
H
C   1  r2
O   2  r3   1   a3
N   2  r4   1   a4   3  d4
H   4  r5   2   a5   3  tau
H   4  r6   2   a6   3  d6
r2   1.0493
r3   1.3000
a3   116.61
r4   1.5385
a4   127.02
d4   184.87
r5   1.0067
a5   109.94
r6   1.0067
a6   108.88
d6   280.53
```

```
tau  160.
$nbo print=0 $end
$DEL
 Lewis
$END
--Link1--
%mem=2gb
%nprocshared=8
%chk=rot_barr
#b3lyp/6-311++g** pop=nbo7del nosymm opt=z-matrix
HCONH2 (tau=170), E(DEL)=-169.761156673 (5.26)
0 1
H
C   1  r2
O   2  r3   1   a3
N   2  r4   1   a4   3  d4
H   4  r5   2   a5   3  tau
H   4  r6   2   a6   3  d6
r2   1.0498
r3   1.2988
a3   116.50
r4   1.5394
a4   127.77
d4   184.84
r5   1.0057
a5   110.03
r6   1.0057
a6   108.73
d6   290.88
tau  170.
$nbo print=0 $end
$DEL
 Lewis
$END
--Link1--
%mem=2gb
%nprocshared=8
%chk=rot_barr
#b3lyp/6-311++g** pop=nbo7del nosymm opt=z-matrix
HCONH2 (tau=180), E(DEL)=-169.761773208 (4.87)
0 1
H
C   1  r2
O   2  r3   1   a3
N   2  r4   1   a4   3  d4
H   4  r5   2   a5   3  tau
H   4  r6   2   a6   3  d6
r2   1.0505
r3   1.2969
a3   116.42
r4   1.5400
a4   128.50
d4   184.19
r5   1.0048
a5   109.84
r6   1.0056
a6   108.56
d6   300.53
tau  180.
$nbo print=0 $end
$DEL
 Lewis
$END
--Link1--
--Link1--
%mem=2gb
%nprocshared=8
%chk=rot_barr
#b3lyp/6-311++g** pop=nbo7read nosymm opt=z-matrix freq
cis-CHFCHF, E(full)=-277.142846266
nu:237,510,771,...
```

```
 Vibrational temperatures:    340.39   733.61  1108.85  1116.07  1251.17
          (Kelvin)           1452.66  1617.65  1842.14  1988.01  2526.05
                             4625.18  4658.01
 Zero-point correction=                           0.036830 (Hartree/Particle)
 Thermal correction to Energy=                    0.040698
 Thermal correction to Enthalpy=                  0.041642
 Thermal correction to Gibbs Free Energy=         0.010510
 Sum of electronic and zero-point Energies=           -277.106017
 Sum of electronic and thermal Energies=              -277.102148
 Sum of electronic and thermal Enthalpies=            -277.101204
 Sum of electronic and thermal Free Energies=         -277.132336
0 1
H
C   1   r2
C   2   r3  1  a3
F   2   r4  3  a4  1  d4
H   3   r5  2  a5  1  d5
F   3   r6  2  a6  4  d6
r2  1.0811
r3  1.3238
a3  122.98
r4  1.3432
a4  122.59
d4  180.00
r5  1.0811
a5  122.99
d5  0.00
r6  1.3432
a6  122.59
d6  0.
$nbo file=cis_chfchf $end
--Link1--
--Link1--
%mem=2gb
%nprocshared=8
%chk=rot_barr
#b3lyp/6-311++g** pop=nbo7del nosymm opt=z-matrix freq
cis-CHFCHF, E(DEL)=-276.972503939
nu:317,334,551,...
 Vibrational temperatures:    456.74   480.82   793.04  1186.31  1301.63
          (Kelvin)           1628.46  1652.72  1851.36  1874.84  2505.74
                             4634.46  4644.87
 Zero-point correction=                           0.036436 (Hartree/Particle)
 Thermal correction to Energy=                    0.040427
 Thermal correction to Enthalpy=                  0.041371
 Thermal correction to Gibbs Free Energy=         0.010285
 Sum of electronic and zero-point Energies=           -277.105129
 Sum of electronic and thermal Energies=              -277.101138
 Sum of electronic and thermal Enthalpies=            -277.100193
 Sum of electronic and thermal Free Energies=         -277.131279
0 1
H
C   1   r2
C   2   r3  1  a3
F   2   r4  3  a4  1  d4
H   3   r5  2  a5  1  d5
F   3   r6  2  a6  4  d6
r2  1.0601
r3  1.3520
a3  133.70
r4  1.4945
a4  115.34
d4  179.97
r5  1.0601
a5  133.70
d5  0.01
r6  1.4946
a6  115.35
d6  -0.02
$nbo print=0 $end
$del
```

```
  lewis
$end
--Link1--
--Link1--
%mem=2gb
%nprocshared=8
%chk=rot_barr
#b3lyp/6-311++g** pop=nbo7read nosymm opt=z-matrix freq
trans-CHFCHF, E(full)=-277.141564470
nu:317,334,551,...
 Vibrational temperatures:    456.74   480.82   793.04  1186.31  1301.63
          (Kelvin)           1628.46  1652.72  1851.36  1874.84  2505.74
                             4634.46  4644.87
 Zero-point correction=                           0.036436 (Hartree/Particle)
 Thermal correction to Energy=                    0.040427
 Thermal correction to Enthalpy=                  0.041371
 Thermal correction to Gibbs Free Energy=         0.010285
 Sum of electronic and zero-point Energies=           -277.105129
 Sum of electronic and thermal Energies=              -277.101138
 Sum of electronic and thermal Enthalpies=            -277.100193
 Sum of electronic and thermal Free Energies=         -277.131279
0 1
H
C   1   r2
C   2   r3  1  a3
F   2   r4  3  a4  1  d4
H   3   r5  2  a5  1  d5
F   3   r6  2  a6  4  d6
r2  1.0814
r3  1.3222
a3  125.71
r4  1.3500
a4  120.03
d4  180.00
r5  1.0814
a5  125.71
d5  180.00
r6  1.3500
a6  120.03
d6  180.00
$nbo file=cis_chfchf $end
--Link1--
--Link1--
%mem=2gb
%nprocshared=8
%chk=rot_barr
#b3lyp/6-311++g** pop=nbo7del nosymm freq
trans-CHFCHF, E(DEL)=-276.979176905
nu: 298,332,478,...
 Vibrational temperatures:    429.09   477.08   688.24  1257.74  1268.76
          (Kelvin)           1506.44  1527.42  1714.19  1776.49  2831.66
                             5012.92  5106.04
 Zero-point correction=                           0.037362 (Hartree/Particle)
 Thermal correction to Energy=                    0.041449
 Thermal correction to Enthalpy=                  0.042394
 Thermal correction to Gibbs Free Energy=         0.010983
 Sum of electronic and zero-point Energies=           -276.941815
 Sum of electronic and thermal Energies=              -276.937727
 Sum of electronic and thermal Enthalpies=            -276.936783
 Sum of electronic and thermal Free Energies=         -276.968194
0 1
H
C   1   r2
C   2   r3  1  a3
F   2   r4  3  a4  1  d4
H   3   r5  2  a5  1  d5
F   3   r6  2  a6  4  d6
r2  1.0621
r3  1.3493
a3  133.44
r4  1.4935
```

```
a4  114.04
d4  179.96
r5  1.0621
a5  133.44
d5  180.06
r6  1.4935
a6  114.01
d6  180.01
$nbo print=0 $end
$del
  lewis
$end
--Link1--
--Link1--
%mem=2gb
%nprocshared=8
%chk=rot_barr
#b3lyp/6-311++g** pop=nbo7read nosymm opt=z-matrix freq
HOOH, E(full)=-151.602169726
nu:365,934,1296,...
 Vibrational temperatures:    525.24  1344.14  1865.30  2090.70  5434.06
          (Kelvin)           5435.42
 Zero-point correction=                           0.026435 (Hartree/Particle)
 Thermal correction to Energy=                    0.029677
 Thermal correction to Enthalpy=                  0.030621
 Thermal correction to Gibbs Free Energy=         0.004121
 Sum of electronic and zero-point Energies=           -151.575735
 Sum of electronic and thermal Energies=              -151.572493
 Sum of electronic and thermal Enthalpies=            -151.571549
 Sum of electronic and thermal Free Energies=         -151.598049
0 1
H
O   1   r2
O   2   r3   1   a3
H   3   r4   2   a4   1   tw
r2   0.9672
r3   1.4540
a3   100.47
r4   0.9672
a4   100.47
tw   121.12
$nbo file=HOOH $end
--Link1--
--Link1--
%mem=2gb
%nprocshared=8
%chk=rot_barr
#b3lyp/6-311++g** pop=nbo7del nosymm freq
HOOH, E(DEL)=-151.577782680
nu: 196,930,1461,...
 Vibrational temperatures:    281.53  1337.88  2101.83  2326.79  5368.40
          (Kelvin)           5422.73
 Zero-point correction=                           0.026663 (Hartree/Particle)
 Thermal correction to Energy=                    0.030120
 Thermal correction to Enthalpy=                  0.031064
 Thermal correction to Gibbs Free Energy=         0.004630
 Sum of electronic and zero-point Energies=           -151.551119
 Sum of electronic and thermal Energies=              -151.547662
 Sum of electronic and thermal Enthalpies=            -151.546718
 Sum of electronic and thermal Free Energies=         -151.573153
0 1
H
O   1   r2
O   2   r3   1   a3
H   3   r4   2   a4   1   tw
r2   0.9720
r3   1.5196
a3   95.41
r4   0.9720
a4   95.41
tw   130.44
```

```
$nbo print=0 $end
$DEL
 Lewis
$END
--Link1--
--Link1--
%mem=2gb
%nprocshared=8
%chk=rot_barr
#b3lyp/6-311++g** pop=nbo7read nosymm opt=modredundant
HOOH rotation barrier
 Summary of Optimized Potential Surface Scan (add -151.0 to energies):
                                 1                  2                  3                  4
5
     Eigenvalues --           -0.588669 (8.47)  -0.588981 (8.28)  -0.589869 (7.72)  -0.591216
(6.87)  -0.592872 (5.83)
           R1                  0.967354           0.967452           0.967627           0.967904
0.968229
           R2                  1.462041           1.462287           1.460884           1.458740
1.456203
           R3                  0.967353           0.967452           0.967627           0.967904
0.968230
           A1                105.323500         105.216869         104.988747         104.649150
104.236775
           A2                105.323472         105.216774         104.989227         104.648880
104.237009
           D1                  0.000000          10.000000          20.000000          30.000000
40.000000
                                 6                  7                  8                  9
10
     Eigenvalues --           -0.594679 (4.70)  -0.596480 (3.57)  -0.598145 (2.53)  -0.599577
(1.63)  -0.600718 (0.91)
           R1                  0.968535           0.968753           0.968830           0.968739
0.968481
           R2                  1.453641           1.451409           1.449799           1.449008
1.449113
           R3                  0.968535           0.968752           0.968830           0.968738
0.968481
           A1                103.785411         103.318931         102.848653         102.373679
101.889558
           A2                103.785464         103.318761         102.848824         102.373432
101.889686
           D1                 50.000000          60.000000          70.000000          80.000000
90.000000
                                11                 12                 13                 14
15
     Eigenvalues --           -0.601531 (0.40)  -0.602005 (0.10)  -0.602168 (0.00)  -0.602089
(0.05)  -0.601855 (0.20)
           R1                  0.968097           0.967661           0.967240           0.966879
0.966598
           R2                  1.450065           1.451713           1.453826           1.456149
1.458426
           R3                  0.968097           0.967661           0.967240           0.966879
0.966598
           A1                101.406120         100.941009         100.509068         100.119859
99.783429
           A2                101.406047         100.941142         100.509274         100.119977
99.783802
           D1                100.000000         110.000000         120.000000         130.000000
140.000000
                                16                 17                 18                 19
     Eigenvalues --           -0.601555 (0.39)  -0.601268 (0.57)  -0.601063 (0.69)  -0.600988
(0.74)
           R1                  0.966402           0.966281           0.966221           0.966223
           R2                  1.460439           1.462008           1.463008           1.463002
           R3                  0.966402           0.966281           0.966221           0.966223
           A1                 99.510911          99.311488          99.191589          99.192705
           A2                 99.510817          99.311853          99.191450          99.192640
           D1                150.000000         160.000000         170.000000         180.000000
0 1
H
```

```
O   1   r2
O   2   r3   1   a3
H   3   r4   2   a4   1   tw
r2   0.9720
r3   1.5196
a3   95.41
r4   0.9720
a4   95.41
tw   0.
1 2 3 4 S 18 10.
$nbo print=0 $end
--Link1--
--Link1--
%mem=2gb
%nprocshared=8
%chk=rot_barr
#b3lyp/6-311++g** pop=nbo7del nosymm opt=z-matrix
HOOH, E(DEL; tau=0)=-151.561164017(NNYY) (10.43)
0 1
H
O   1   r2
O   2   r3   1   a3
H   3   r4   2   a4   1   tw
r2   0.9749
r3   1.5312
a3   99.94
r4   0.9749
a4   99.94
tw   0.
$nbo print=0 $end
$DEL
 Lewis
$END
--Link1--
%mem=2gb
%nprocshared=8
%chk=rot_barr
#b3lyp/6-311++g** pop=nbo7del nosymm opt=z-matrix
HOOH, E(DEL; tau=10)=-151.561484852(YYYY) (10.23)
0 1
H
O   1   r2
O   2   r3   1   a3
H   3   r4   2   a4   1   tw
r2   0.9762
r3   1.5294
a3   99.85
r4   0.9762
a4   99.85
tw   10.
$nbo print=0 $end
$DEL
 Lewis
$END
--Link1--
%mem=2gb
%nprocshared=8
%chk=rot_barr
#b3lyp/6-311++g** pop=nbo7del nosymm opt=z-matrix
HOOH, E(DEL; tau=20)=-151.562428514(NNYY) (9.63)
0 1
H
O   1   r2
O   2   r3   1   a3
H   3   r4   2   a4   1   tw
r2   0.9767
r3   1.5269
a3   99.79
r4   0.9767
a4   99.82
tw   20.
```

```
$nbo print=0 $end
$DEL
 Lewis
$END
--Link1--
%mem=2gb
%nprocshared=8
%chk=rot_barr
#b3lyp/6-311++g** pop=nbo7del nosymm opt=z-matrix
HOOH, E(DEL; tau=30)=-151.563970462(YYYY) (8.67)
0 1
H
O   1   r2
O   2   r3   1   a3
H   3   r4   2   a4   1   tw
r2   0.9739
r3   1.5272
a3   98.91
r4   0.9739
a4   98.91
tw   30.
$nbo print=0 $end
$DEL
 Lewis
$END
--Link1--
%mem=2gb
%nprocshared=8
%chk=rot_barr
#b3lyp/6-311++g** pop=nbo7del nosymm opt=z-matrix
HOOH, E(DEL; tau=40)=-151.565871693(YYYY) (7.47)
0 1
H
O   1   r2
O   2   r3   1   a3
H   3   r4   2   a4   1   tw
r2   0.9739
r3   1.5254
a3   98.31
r4   0.9739
a4   98.31
tw   40.
$nbo print=0 $end
$DEL
 Lewis
$END
--Link1--
%mem=2gb
%nprocshared=8
%chk=rot_barr
#b3lyp/6-311++g** pop=nbo7del nosymm opt=z-matrix
HOOH, E(DEL; tau=50)=-151.567965276 (6.16)
0 1
H
O   1   r2
O   2   r3   1   a3
H   3   r4   2   a4   1   tw
r2   0.9739
r3   1.5236
a3   97.69
r4   0.9739
a4   97.69
tw   50.
$nbo print=0 $end
$DEL
 Lewis
$END
--Link1--
%mem=2gb
%nprocshared=8
%chk=rot_barr
```

```
#b3lyp/6-311++g** pop=nbo7del nosymm opt=z-matrix
HOOH, E(DEL; tau=60)=-151.570068697 (4.84)
0 1
H
O   1   r2
O   2   r3   1   a3
H   3   r4   2   a4   1   tw
r2   0.9741
r3   1.5229
a3   97.08
r4   0.9741
a4   97.08
tw   60.
$nbo print=0 $end
$DEL
 Lewis
$END
--Link1--
%mem=2gb
%nprocshared=8
%chk=rot_barr
#b3lyp/6-311++g** pop=nbo7del nosymm opt=z-matrix
HOOH, E(DEL; tau=70)=-151.572041240 (3.60)
0 1
H
O   1   r2
O   2   r3   1   a3
H   3   r4   2   a4   1   tw
r2   0.9740
r3   1.5212
a3   96.60
r4   0.9740
a4   96.60
tw   70.
$nbo print=0 $end
$DEL
 Lewis
$END
--Link1--
%mem=2gb
%nprocshared=8
%chk=rot_barr
#b3lyp/6-311++g** pop=nbo7del nosymm opt=z-matrix
HOOH, E(DEL; tau=80)=-151.573787126 (2.51)
0 1
H
O   1   r2
O   2   r3   1   a3
H   3   r4   2   a4   1   tw
r2   0.9739
r3   1.5200
a3   96.21
r4   0.9739
a4   96.21
tw   80.
$nbo print=0 $end
$DEL
 Lewis
$END
--Link1--
%mem=2gb
%nprocshared=8
%chk=rot_barr
#b3lyp/6-311++g** pop=nbo7del nosymm opt=z-matrix
HOOH, E(DEL; tau=90)=-151.575261231 (1.58)
0 1
H
O   1   r2
O   2   r3   1   a3
H   3   r4   2   a4   1   tw
r2   0.9736
```

```
r3   1.5192
a3   95.92
r4   0.9736
a4   95.92
tw   90.
$nbo print=0 $end
$DEL
 Lewis
$END
--Link1--
%mem=2gb
%nprocshared=8
%chk=rot_barr
#b3lyp/6-311++g** pop=nbo7del nosymm opt=z-matrix
HOOH, E(DEL; tau=100)= -151.576397011 (0.87)
0 1
H
O   1   r2
O   2   r3   1   a3
H   3   r4   2   a4   1   tw
r2   0.9733
r3   1.5195
a3   95.69
r4   0.9733
a4   95.69
tw   100.
$nbo print=0 $end
$DEL
 Lewis
$END
--Link1--
%mem=2gb
%nprocshared=8
%chk=rot_barr
#b3lyp/6-311++g** pop=nbo7del nosymm opt=z-matrix
HOOH, E(DEL; tau=110)=-151.577189565 (0.37)
0 1
H
O   1   r2
O   2   r3   1   a3
H   3   r4   2   a4   1   tw
r2   0.9728
r3   1.5190
a3   95.52
r4   0.9728
a4   95.52
tw   110.
$nbo print=0 $end
$DEL
 Lewis
$END
--Link1--
%mem=2gb
%nprocshared=8
%chk=rot_barr
#b3lyp/6-311++g** pop=nbo7del nosymm opt=z-matrix
HOOH, E(DEL; tau=120)=-151.577639459 (0.09)
0 1
H
O   1   r2
O   2   r3   1   a3
H   3   r4   2   a4   1   tw
r2   0.9720
r3   1.5196
a3   95.41
r4   0.9720
a4   95.41
tw   120.
$nbo print=0 $end
$DEL
 Lewis
```

```
$END
--Link1--
%mem=2gb
%nprocshared=8
%chk=rot_barr
#b3lyp/6-311++g** pop=nbo7del nosymm opt=z-matrix
HOOH, E(DEL; tau=130)=-151.577782480 (0.00)
0 1
H
O   1   r2
O   2   r3   1   a3
H   3   r4   2   a4   1   tw
r2   0.9720
r3   1.5196
a3   95.41
r4   0.9720
a4   95.41
tw   130.
$nbo print=0 $end
$DEL
 Lewis
$END
--Link1--
%mem=2gb
%nprocshared=8
%chk=rot_barr
#b3lyp/6-311++g** pop=nbo7del nosymm opt=z-matrix
HOOH, E(DEL; tau=140)=-151.577686248 (0.06)
0 1
H
O   1   r2
O   2   r3   1   a3
H   3   r4   2   a4   1   tw
r2   0.9720
r3   1.5196
a3   95.41
r4   0.9720
a4   95.41
tw   140.
$nbo print=0 $end
$DEL
 Lewis
$END
--Link1--
%mem=2gb
%nprocshared=8
%chk=rot_barr
#b3lyp/6-311++g** pop=nbo7del nosymm opt=z-matrix
HOOH, E(DEL; tau=150)=-151.577450750 (0.21)
0 1
H
O   1   r2
O   2   r3   1   a3
H   3   r4   2   a4   1   tw
r2   0.9714
r3   1.5212
a3   95.54
r4   0.9714
a4   95.54
tw   150.
$nbo print=0 $end
$DEL
 Lewis
$END
--Link1--
%mem=2gb
%nprocshared=8
%chk=rot_barr
#b3lyp/6-311++g** pop=nbo7del nosymm opt=z-matrix
HOOH, E(DEL; tau=160)=-151.577183862 (0.38)
0 1
```

```
H
O   1   r2
O   2   r3   1   a3
H   3   r4   2   a4   1   tw
r2   0.9712
r3   1.5214
a3   95.58
r4   0.9712
a4   95.58
tw   160.
$nbo print=0 $end
$DEL
 Lewis
$END
--Link1--
%mem=2gb
%nprocshared=8
%chk=rot_barr
#b3lyp/6-311++g** pop=nbo7del nosymm opt=z-matrix
HOOH, E(DEL; tau=170)=-151.576981609 (0.50)
0 1
H
O   1   r2
O   2   r3   1   a3
H   3   r4   2   a4   1   tw
r2   0.9711
r3   1.5219
a3   95.64
r4   0.9711
a4   95.64
tw   170.
$nbo print=0 $end
$DEL
 Lewis
$END
--Link1--
%mem=2gb
%nprocshared=8
%chk=rot_barr
#b3lyp/6-311++g** pop=nbo7del nosymm opt=z-matrix
HOOH, E(DEL; tau=180)=-151.576907220 (0.55)
0 1
H
O   1   r2
O   2   r3   1   a3
H   3   r4   2   a4   1   tw
r2   0.9711
r3   1.5222
a3   95.66
r4   0.9711
a4   95.66
tw   180.
$nbo print=0 $end
$DEL
 Lewis
$END
--Link1--
--Link1--
%mem=2gb
%nprocshared=8
%chk=rot_barr
#b3lyp/6-311++g** pop=nbo7read nosymm opt=z-matrix freq
CH3CH3, E(full)=-79.8565752846
nu: 307,827(2),...
 Vibrational temperatures:    442.41  1189.83  1189.97  1432.90  1751.90
          (Kelvin)           1751.96  2026.18  2047.75  2162.85  2162.97
                             2165.96  2166.09  4350.46  4350.69  4416.15
                             4416.26  4452.09  4452.20
 Zero-point correction=                           0.074307 (Hartree/Particle)
 Thermal correction to Energy=                    0.077794
 Thermal correction to Enthalpy=                  0.078738
```

```
 Thermal correction to Gibbs Free Energy=         0.051189
 Sum of electronic and zero-point Energies=           -79.782268
 Sum of electronic and thermal Energies=              -79.778781
 Sum of electronic and thermal Enthalpies=            -79.777837
 Sum of electronic and thermal Free Energies=         -79.805386
0 1
C
C   1   r2
H   1   r3   2  a3
H   1   r4   2  a4   3  d4
H   1   r5   2  a5   4  d5
H   2   r6   1  a6   3  tw
H   2   r7   1  a7   6  d7
H   2   r8   1  a8   7  d8
r2  1.5309
r3  1.0937
a3  111.36
r4  1.0937
a4  111.35
d4  119.98
r5  1.0937
a5  111.36
d5  120.01
r6  1.0937
a6  111.36
tw  60.12
r7  1.0937
a7  111.35
d7  119.98
r8  1.0937
a8  111.36
d8  120.01
$nbo file=ch3ch3 $end
--Link1--
--Link1--
%mem=2gb
%nprocshared=8
%chk=rot_barr
#b3lyp/6-311++g** nosymm opt=(ts,z-matrix,calcfc) freq
CH3CH3 (eclipsed t.s.), -79.8522677640
nu: -299,896(2),991,...
 Vibrational temperatures:   1288.47  1288.52  1426.25  1683.22  1683.24
          (Kelvin)           2023.87  2069.64  2160.67  2160.73  2173.64
                             2173.69  4361.51  4371.92  4429.42  4429.46
                             4462.17  4462.21
 Zero-point correction=                           0.073864 (Hartree/Particle)
 Thermal correction to Energy=                    0.076915
 Thermal correction to Enthalpy=                  0.077859
 Thermal correction to Gibbs Free Energy=         0.050985
 Sum of electronic and zero-point Energies=           -79.778404
 Sum of electronic and thermal Energies=              -79.775353
 Sum of electronic and thermal Enthalpies=            -79.774409
 Sum of electronic and thermal Free Energies=         -79.801283
0 1
C
C   1   r2
H   1   r3   2  a3
H   1   r4   2  a4   3  d4
H   1   r5   2  a5   4  d5
H   2   r6   1  a6   3  tw
H   2   r7   1  a7   6  d7
H   2   r8   1  a8   7  d8
r2  1.5447
r3  1.0925
a3  111.79
r4  1.0925
a4  111.79
d4  120.00
r5  1.0925
a5  111.79
d5  120.00
```

```
r6  1.0925
a6  111.79
r7  1.0925
a7  111.79
d7  120.00
r8  1.0925
a8  111.79
d8  120.00
tw  0.
$nbo file=ch3ch3_ecl archive nrt plot steric $end
--Link1--
--Link1--
%mem=2gb
%nprocshared=8
%chk=rot_barr
#b3lyp/6-311++g** pop=nbo7del nosymm freq
CH3CH3, E(DEL)=-79.817928022 (YYYY) (0.00)
nu: 29,918(2),938,1219(2),1475,1518,1599(2),1602(2),3130(2),3144(2),3253,3284
 Vibrational temperatures:     41.86  1320.25  1320.34  1350.26  1753.47
          (Kelvin)           1753.50  2122.29  2184.01  2299.29  2299.97
                             2305.30  2305.98  4503.55  4503.77  4523.09
                             4523.32  4681.05  4725.22
 Zero-point correction=                           0.076821 (Hartree/Particle)
 Thermal correction to Energy=                    0.080735
 Thermal correction to Enthalpy=                  0.081679
 Thermal correction to Gibbs Free Energy=         0.051902
 Sum of electronic and zero-point Energies=            -79.741107
 Sum of electronic and thermal Energies=               -79.737193
 Sum of electronic and thermal Enthalpies=             -79.736249
 Sum of electronic and thermal Free Energies=          -79.766026
0 1
C
C   1   r2
H   1   r3   2  a3
H   1   r4   2  a4   3  d4
H   1   r5   2  a5   4  d5
H   2   r6   1  a6   3  tw
H   2   r7   1  a7   6  d7
H   2   r8   1  a8   7  d8
r2  1.6826
r3  1.0928
a3  109.74
r4  1.0928
a4  109.74
d4  120.00
r5  1.0928
a5  109.74
d5  120.00
r6  1.0928
a6  109.74
tw  63.26
r7  1.0928
a7  109.74
d7  119.99
r8  1.0928
a8  109.74
d8  120.00

$nbo print=0 $end
$DEL
  Lewis
$END
--Link1--
--Link1--
%mem=2gb
%nprocshared=8
%chk=rot_barr
#b3lyp/6-311++g** nosymm opt=modredundant
CH3CH3 barrier, E(full)
 Summary of Optimized Potential Surface Scan (add -79.0 to energies):
```

	1	2	3	4	5
Eigenvalues --	-0.852268 (2.70)	-0.852584 (2.50)	-0.853423 (1.98)	-0.854520 (1.29)	-0.855569 (0.63)
R1	1.544148	1.543269	1.540409	1.536779	1.533572
R2	1.092586	1.092665	1.092813	1.093072	1.093356
R3	1.092587	1.092230	1.092139	1.092399	1.092801
R4	1.092586	1.093070	1.093659	1.094069	1.094188
R5	1.092586	1.092665	1.092813	1.093072	1.093356
R6	1.092587	1.092230	1.092139	1.092399	1.092801
R7	1.092586	1.093070	1.093659	1.094069	1.094188
A1	111.806253	111.758520	111.649178	111.518526	111.407470
A2	111.805871	112.046617	112.131997	112.068873	111.876239
A3	111.805901	111.496508	111.229159	111.085747	111.073808
A4	107.038763	107.717215	108.234283	108.460842	108.374103
A5	107.038943	106.435767	106.097952	106.126374	106.445440
A6	107.038906	107.080920	107.201209	107.304672	107.419792
A7	111.806253	111.758520	111.649177	111.518522	111.407466
A8	111.805860	112.046619	112.132005	112.068874	111.876241
A9	111.805911	111.496505	111.229154	111.085751	111.073806
A10	107.038789	107.717212	108.234259	108.460847	108.374101
A11	107.038916	106.435769	106.097975	106.126368	106.445443
A12	107.038906	107.080921	107.201207	107.304671	107.419794
D1	-0.000009	10.000183	20.000306	30.000412	40.000308
D2	119.999953	131.007720	141.659087	151.813138	161.484589
D3	-120.000171	-108.987028	-98.308963	-88.165206	-78.485998
D4	119.999927	131.007722	141.659112	151.813133	161.484593
D5	-120.000110	-107.984742	-96.682107	-86.374142	-77.031126
D6	-0.000234	12.020510	23.349843	33.647515	42.998287
D7	-120.000197	-108.987027	-98.308937	-88.165213	-78.485997
D8	-0.000234	12.020510	23.349844	33.647512	42.998284
D9	119.999641	132.025762	143.381794	153.669169	163.027697

	6	7	8	9	10
Eigenvalues --	-0.856309 (0.17)	-0.856575 (0.00)	-0.856309 (0.17)	-0.855567 (0.63)	-0.854517 (1.29)
R1	1.531433	1.530681	1.531412	1.533534	1.536717
R2	1.093561	1.093681	1.093639	1.093497	1.093221
R3	1.093275	1.093706	1.094028	1.094122	1.093982

R4	1.094047	1.093690	1.093236	1.092755	1.092377
R5	1.093561	1.093681	1.093639	1.093497	1.093221
R6	1.093275	1.093706	1.094028	1.094122	1.093982
R7	1.094047	1.093690	1.093236	1.092755	1.092377
A1	111.338211	111.355649	111.424669	111.547763	111.644941
A2	111.624781	111.345250	111.127259	110.991706	111.028661
A3	111.176512	111.361326	111.589961	111.826185	112.013305
A4	108.033110	107.523392	106.958091	106.455977	106.146932
A5	106.955006	107.512501	108.015078	108.327345	108.398476
A6	107.497109	107.537608	107.509527	107.447485	107.332900
A7	111.338208	111.355645	111.424672	111.547763	111.644952
A8	111.624783	111.345248	111.127260	110.991693	111.028662
A9	111.176517	111.361323	111.589972	111.826176	112.013318
A10	108.033122	107.523391	106.958102	106.455973	106.146932
A11	106.954991	107.512507	108.015058	108.327362	108.398460
A12	107.497106	107.537613	107.509520	107.447495	107.332889
D1	50.000251	60.000347	70.000368	80.000106	90.000279
D2	170.822613	179.994025	-170.821384	-161.462896	-151.763633
D3	-69.149591	-59.990399	-50.834872	-41.488333	-31.783080
D4	170.822598	179.994031	-170.821400	-161.462883	-151.763641
D5	-68.355040	-60.012291	-51.643152	-42.925885	-33.527553
D6	51.672756	60.003285	68.343359	77.048679	86.452999
D7	-69.149608	-59.990396	-50.834888	-41.488317	-31.783084
D8	51.672753	60.003282	68.343360	77.048681	86.453003
D9	171.700550	-179.981142	-171.670128	-162.976755	-153.566444

	11	12	13	14	15
Eigenvalues --	-0.853420 (1.98)	-0.852582 (2.51)	-0.852268 (2.70)	-0.852583 (2.51)	-0.853422 (1.98)
R1	1.540314	1.543267	1.544452	1.543318	1.540365
R2	1.092941	1.092670	1.092556	1.092600	1.092818
R3	1.093556	1.093023	1.092515	1.092256	1.092266
R4	1.092170	1.092224	1.092539	1.093037	1.093550
R5	1.092941	1.092670	1.092556	1.092600	1.092818
R6	1.093556	1.093023	1.092515	1.092256	1.092266
R7	1.092169	1.092224	1.092539	1.093037	1.093550
A1	111.725507	111.757957	111.766122	111.743338	111.679394

A2	111.200553	111.499814	111.810451	112.056826	112.169238
A3	112.107349	112.042053	111.820366	111.487735	111.167237
A4	106.132485	106.456783	107.040433	107.694986	108.201317
A5	108.151903	107.658630	107.024061	106.467789	106.157335
A6	107.225764	107.121207	107.074902	107.086248	107.168341
A7	111.725503	111.757959	111.766112	111.743338	111.679392
A8	111.200544	111.499831	111.810463	112.056851	112.169253
A9	112.107335	112.042058	111.820349	111.487724	111.167217
A10	106.132474	106.456763	107.040410	107.694963	108.201309
A11	108.151932	107.658634	107.024093	106.467803	106.157350
A12	107.225774	107.121198	107.074910	107.086241	107.168341
D1	100.000227	110.000265	120.000334	130.000335	140.000309
D2	-141.615909	-130.984015	-120.022244	-109.024410	-98.335043
D3	-21.589281	-10.928034	0.036961	10.988593	21.637372
D4	-141.615888	-130.984002	-120.022215	-109.024398	-98.335042
D5	-23.232024	-11.968283	-0.044793	11.950856	23.329607
D6	96.794604	108.087699	120.014412	131.963859	143.302021
D7	-21.589257	-10.928024	0.036984	10.988604	21.637376
D8	96.794607	108.087696	120.014406	131.963859	143.302024
D9	-143.178765	-131.856323	-119.926389	-108.023138	-96.725561

	16	17	18	19
Eigenvalues --	-0.854521 (1.29)	-0.855570 (0.63)	-0.856310 (0.17)	-0.856575 (0.00)
R1	1.536752	1.533546	1.531410	1.530662
R2	1.093112	1.093402	1.093598	1.093664
R3	1.092516	1.092892	1.093330	1.093740
R4	1.093919	1.094055	1.093960	1.093687
R5	1.093112	1.093402	1.093598	1.093664
R6	1.092516	1.092892	1.093331	1.093741
R7	1.093919	1.094055	1.093960	1.093686
A1	111.577630	111.463295	111.371380	111.328778
A2	112.133656	111.953155	111.678861	111.373092
A3	110.964690	110.944481	111.096256	111.373554
A4	108.423435	108.342536	108.011031	107.518058
A5	106.176184	106.480607	106.969426	107.513786
A6	107.286887	107.410030	107.496347	107.527564
A7	111.577633	111.463293	111.371387	111.328785
A8	112.133678	111.953178	111.678903	111.373140
A9	110.964673	110.944455	111.096220	111.373495
A10	108.423432	108.342543	108.011038	107.518073
A11	106.176182	106.480604	106.969411	107.513774
A12	107.286882	107.410031	107.496340	107.527565
D1	150.000230	160.000329	170.000247	-179.999769
D2	-88.146321	-78.461885	-69.145089	-60.012148
D3	31.812980	41.518322	50.863561	60.017826
D4	-88.146336	-78.461910	-69.145132	-60.012204
D5	33.707113	43.075875	51.709531	59.975417
D6	153.666414	163.056083	171.718182	-179.994609
D7	31.812968	41.518300	50.863523	60.017776
D8	153.666417	163.056085	171.718186	-179.994603
D9	-86.374282	-76.963708	-68.273163	-59.964629

```
0 1
C
C   1   r2
H   1   r3   2  a3
H   1   r4   2  a4   3  d4
H   1   r5   2  a5   4  d5
H   2   r6   1  a6   3  tw
H   2   r7   1  a7   6  d7
H   2   r8   1  a8   7  d8
r2  1.6826
r3  1.0928
a3  109.74
r4  1.0928
a4  109.74
d4  120.00
r5  1.0928
a5  109.74
d5  120.00
r6  1.0928
a6  109.74
r7  1.0928
a7  109.74
d7  119.99
r8  1.0928
a8  109.74
d8  120.00
tw  0.

3 1 2 6 S 18 10.
--Link1--
%mem=2gb
%nprocshared=8
%chk=rot_barr
#b3lyp/6-311++g** pop=nbo7del nosymm opt=z-matrix
CH3CH3 barrier, E(DEL), tau=0,-79.817850906 (0.05)
0 1
C
C   1   r2
H   1   r3   2  a3
H   1   r4   2  a4   3  d4
H   1   r5   2  a5   4  d5
H   2   r6   1  a6   3  tw
H   2   r7   1  a7   6  d7
H   2   r8   1  a8   7  d8
r2  1.6775
r3  1.0929
a3  109.67
r4  1.0929
a4  109.67
d4  120.00
r5  1.0929
a5  109.67
d5  120.00
r6  1.0929
a6  109.67
r7  1.0929
a7  109.67
d7  120.00
r8  1.0929
a8  109.67
d8  120.00
tw  0.

$nbo print=0 $end
$DEL
  Lewis
$END
--Link1--
%mem=2gb
%nprocshared=8
%chk=rot_barr
```

```
#b3lyp/6-311++g** pop=nbo7del nosymm opt=z-matrix
CH3CH3 barrier, E(DEL), tau=10,-79.817857087 (0.04)
0 1
C
C   1   r2
H   1   r3   2  a3
H   1   r4   2  a4   3  d4
H   1   r5   2  a5   4  d5
H   2   r6   1  a6   3  tw
H   2   r7   1  a7   6  d7
H   2   r8   1  a8   7  d8
r2  1.6778
r3  1.0929
a3  109.67
r4  1.0929
a4  109.68
d4  120.01
r5  1.0929
a5  109.67
d5  120.00
r6  1.0929
a6  109.67
r7  1.0929
a7  109.68
d7  120.01
r8  1.0929
a8  109.67
d8  120.00
tw  10.

$nbo print=0 $end
$DEL
  Lewis
$END
--Link1--
%mem=2gb
%nprocshared=8
%chk=rot_barr
#b3lyp/6-311++g** pop=nbo7del nosymm opt=z-matrix
CH3CH3 barrier, E(DEL), tau=20,-79.817873243 (0.03)
0 1
C
C   1   r2
H   1   r3   2  a3
H   1   r4   2  a4   3  d4
H   1   r5   2  a5   4  d5
H   2   r6   1  a6   3  tw
H   2   r7   1  a7   6  d7
H   2   r8   1  a8   7  d8
r2  1.6788
r3  1.0929
a3  109.68
r4  1.0928
a4  109.69
d4  120.02
r5  1.0929
a5  109.68
d5  120.00
r6  1.0929
a6  109.68
r7  1.0928
a7  109.69
d7  120.02
r8  1.0929
a8  109.68
d8  120.00
tw  20.

$nbo print=0 $end
$DEL
  Lewis
```

```
$END
--Link1--
%mem=2gb
%nprocshared=8
%chk=rot_barr
#b3lyp/6-311++g** pop=nbo7del nosymm opt=z-matrix
CH3CH3 barrier, E(DEL), tau=30,-79.817892776 (0.02)
0 1
C
C   1   r2
H   1   r3   2  a3
H   1   r4   2  a4   3  d4
H   1   r5   2  a5   4  d5
H   2   r6   1  a6   3  tw
H   2   r7   1  a7   6  d7
H   2   r8   1  a8   7  d8
r2  1.6798
r3  1.0928
a3  109.66
r4  1.0928
a4  109.66
d4  120.03
r5  1.0928
a5  109.66
d5  120.01
r6  1.0928
a6  109.66
r7  1.0928
a7  109.65
d7  120.03
r8  1.0928
a8  109.66
d8  120.02
tw  30.

$nbo print=0 $end
$DEL
  Lewis
$END
--Link1--
%mem=2gb
%nprocshared=8
%chk=rot_barr
#b3lyp/6-311++g** pop=nbo7del nosymm opt=z-matrix
CH3CH3 barrier, E(DEL), tau=40,-79.817850906 (0.05)
0 1
C
C   1   r2
H   1   r3   2  a3
H   1   r4   2  a4   3  d4
H   1   r5   2  a5   4  d5
H   2   r6   1  a6   3  tw
H   2   r7   1  a7   6  d7
H   2   r8   1  a8   7  d8
r2  1.6775
r3  1.0929
a3  109.67
r4  1.0929
a4  109.67
d4  120.00
r5  1.0929
a5  109.67
d5  120.00
r6  1.0929
a6  109.67
r7  1.0929
a7  109.67
d7  120.00
r8  1.0929
a8  109.67
d8  120.00
```

```
tw  40.

$nbo print=0 $end
$DEL
  Lewis
$END
--Link1--
%mem=2gb
%nprocshared=8
%chk=rot_barr
#b3lyp/6-311++g** pop=nbo7del nosymm opt=z-matrix
CH3CH3 barrier, E(DEL), tau=50,-79.817924097 (0.00)
0 1
C
C   1   r2
H   1   r3   2  a3
H   1   r4   2  a4   3  d4
H   1   r5   2  a5   4  d5
H   2   r6   1  a6   3  tw
H   2   r7   1  a7   6  d7
H   2   r8   1  a8   7  d8
r2  1.6826
r3  1.0928
a3  109.74
r4  1.0928
a4  109.74
d4  120.00
r5  1.0928
a5  109.74
d5  120.00
r6  1.0928
a6  109.74
r7  1.0928
a7  109.74
d7  119.99
r8  1.0928
a8  109.74
d8  120.00
tw  50.

$nbo print=0 $end
$DEL
  Lewis
$END
--Link1--
%mem=2gb
%nprocshared=8
%chk=rot_barr
#b3lyp/6-311++g** pop=nbo7del nosymm opt=z-matrix
CH3CH3 barrier, E(DEL), tau=60,-79.817928455 (0.00)
0 1
C
C   1   r2
H   1   r3   2  a3
H   1   r4   2  a4   3  d4
H   1   r5   2  a5   4  d5
H   2   r6   1  a6   3  tw
H   2   r7   1  a7   6  d7
H   2   r8   1  a8   7  d8
r2  1.6826
r3  1.0928
a3  109.74
r4  1.0928
a4  109.74
d4  120.00
r5  1.0928
a5  109.74
d5  120.00
r6  1.0928
a6  109.74
r7  1.0928
```

```
a7  109.74
d7  119.99
r8  1.0928
a8  109.74
d8  120.00
tw  60.

$nbo print=0 $end
$DEL
  Lewis
$END
--Link1--
%mem=2gb
%nprocshared=8
%chk=rot_barr
#b3lyp/6-311++g** pop=nbo7del nosymm opt=z-matrix
CH3CH3 barrier, E(DEL), tau=70,-79.817924132 (0.00)
0 1
C
C   1   r2
H   1   r3   2  a3
H   1   r4   2  a4   3  d4
H   1   r5   2  a5   4  d5
H   2   r6   1  a6   3  tw
H   2   r7   1  a7   6  d7
H   2   r8   1  a8   7  d8
r2  1.6826
r3  1.0928
a3  109.74
r4  1.0928
a4  109.74
d4  120.00
r5  1.0928
a5  109.74
d5  120.00
r6  1.0928
a6  109.74
r7  1.0928
a7  109.74
d7  119.99
r8  1.0928
a8  109.74
d8  120.00
tw  70.

$nbo print=0 $end
$DEL
  Lewis
$END
--Link1--
%mem=2gb
%nprocshared=8
%chk=rot_barr
#b3lyp/6-311++g** pop=nbo7del nosymm opt=z-matrix
CH3CH3 barrier, E(DEL), tau=80,-79.817911711 (0.01)
0 1
C
C   1   r2
H   1   r3   2  a3
H   1   r4   2  a4   3  d4
H   1   r5   2  a5   4  d5
H   2   r6   1  a6   3  tw
H   2   r7   1  a7   6  d7
H   2   r8   1  a8   7  d8
r2  1.6812
r3  1.0928
a3  109.70
r4  1.0928
a4  109.70
d4  119.98
r5  1.0928
```

```
a5  109.70
d5  119.99
r6  1.0928
a6  109.70
r7  1.0928
a7  109.70
d7  119.98
r8  1.0928
a8  109.70
d8  120.00
tw  80.

$nbo print=0 $end
$DEL
  Lewis
$END
--Link1--
%mem=2gb
%nprocshared=8
%chk=rot_barr
#b3lyp/6-311++g** pop=nbo7del nosymm opt=z-matrix
CH3CH3 barrier, E(DEL), tau=90, -79.817892715 (0.02)
0 1
C
C   1   r2
H   1   r3   2  a3
H   1   r4   2  a4   3  d4
H   1   r5   2  a5   4  d5
H   2   r6   1  a6   3  tw
H   2   r7   1  a7   6  d7
H   2   r8   1  a8   7  d8
r2  1.6798
r3  1.0928
a3  109.66
r4  1.0928
a4  109.66
d4  119.97
r5  1.0928
a5  109.66
d5  119.99
r6  1.0928
a6  109.66
r7  1.0928
a7  109.65
d7  119.97
r8  1.0928
a8  109.66
d8  119.99
tw  90.

$nbo print=0 $end
$DEL
  Lewis
$END
--Link1--
%mem=2gb
%nprocshared=8
%chk=rot_barr
#b3lyp/6-311++g** pop=nbo7del nosymm opt=z-matrix
CH3CH3 barrier, E(DEL), tau=100,-79.817873261 (0.03)
0 1
C
C   1   r2
H   1   r3   2  a3
H   1   r4   2  a4   3  d4
H   1   r5   2  a5   4  d5
H   2   r6   1  a6   3  tw
H   2   r7   1  a7   6  d7
H   2   r8   1  a8   7  d8
r2  1.6788
r3  1.0929
```

```
a3  109.70
r4  1.0929
a4  109.68
d4  119.98
r5  1.0928
a5  109.69
d5  120.00
r6  1.0929
a6  109.69
r7  1.0929
a7  109.68
d7  119.98
r8  1.0928
a8  109.69
d8  120.00
tw  100.

$nbo print=0 $end
$DEL
  Lewis
$END
--Link1--
%mem=2gb
%nprocshared=8
%chk=rot_barr
#b3lyp/6-311++g** pop=nbo7del nosymm opt=z-matrix
CH3CH3 barrier, E(DEL), tau=110,-79.817857100 (0.04)
0 1
C
C   1   r2
H   1   r3   2  a3
H   1   r4   2  a4   3  d4
H   1   r5   2  a5   4  d5
H   2   r6   1  a6   3  tw
H   2   r7   1  a7   6  d7
H   2   r8   1  a8   7  d8
r2  1.6778
r3  1.0929
a3  109.68
r4  1.0929
a4  109.67
d4  119.99
r5  1.0929
a5  109.68
d5  120.00
r6  1.0929
a6  109.68
r7  1.0929
a7  109.67
d7  119.99
r8  1.0929
a8  109.68
d8  120.00
tw  110.

$nbo print=0 $end
$DEL
  Lewis
$END
--Link1--
%mem=2gb
%nprocshared=8
%chk=rot_barr
#b3lyp/6-311++g** pop=nbo7del nosymm opt=z-matrix
CH3CH3 barrier, E(DEL), tau=120,-79.817850906 (0.05)
0 1
C
C   1   r2
H   1   r3   2  a3
H   1   r4   2  a4   3  d4
H   1   r5   2  a5   4  d5
```

```
H   2   r6   1  a6   3  tw
H   2   r7   1  a7   6  d7
H   2   r8   1  a8   7  d8
r2  1.6775
r3  1.0929
a3  109.67
r4  1.0929
a4  109.67
d4  120.00
r5  1.0929
a5  109.67
d5  120.00
r6  1.0929
a6  109.67
r7  1.0929
a7  109.67
d7  120.00
r8  1.0929
a8  109.67
d8  120.00
tw  120.

$nbo print=0 $end
$DEL
  Lewis
$END
--Link1--
%mem=2gb
%nprocshared=8
%chk=rot_barr
#b3lyp/6-311++g** pop=nbo7del nosymm opt=z-matrix
CH3CH3 barrier, E(DEL), tau=130,-79.817857086 (0.04)
0 1
C
C   1   r2
H   1   r3   2  a3
H   1   r4   2  a4   3  d4
H   1   r5   2  a5   4  d5
H   2   r6   1  a6   3  tw
H   2   r7   1  a7   6  d7
H   2   r8   1  a8   7  d8
r2  1.6778
r3  1.0929
a3  109.67
r4  1.0929
a4  109.68
d4  120.01
r5  1.0929
a5  109.67
d5  120.00
r6  1.0929
a6  109.67
r7  1.0929
a7  109.68
d7  120.01
r8  1.0929
a8  109.67
d8  120.00
tw  130.

$nbo print=0 $end
$DEL
  Lewis
$END
--Link1--
%mem=2gb
%nprocshared=8
%chk=rot_barr
#b3lyp/6-311++g** pop=nbo7del nosymm opt=z-matrix
CH3CH3 barrier, E(DEL), tau=140,-79.817873244 (0.03)
0 1
```

```
C
C   1   r2
H   1   r3   2  a3
H   1   r4   2  a4   3  d4
H   1   r5   2  a5   4  d5
H   2   r6   1  a6   3  tw
H   2   r7   1  a7   6  d7
H   2   r8   1  a8   7  d8
r2  1.6788
r3  1.0929
a3  109.68
r4  1.0928
a4  109.69
d4  120.02
r5  1.0929
a5  109.68
d5  120.00
r6  1.0929
a6  109.68
r7  1.0928
a7  109.69
d7  120.02
r8  1.0928
a8  109.68
d8  120.00
tw  140.

$nbo print=0 $end
$DEL
  Lewis
$END
--Link1--
%mem=2gb
%nprocshared=8
%chk=rot_barr
#b3lyp/6-311++g** pop=nbo7del nosymm opt=z-matrix
CH3CH3 barrier, E(DEL), tau=150,-79.817892775 (0.02)
0 1
C
C   1   r2
H   1   r3   2  a3
H   1   r4   2  a4   3  d4
H   1   r5   2  a5   4  d5
H   2   r6   1  a6   3  tw
H   2   r7   1  a7   6  d7
H   2   r8   1  a8   7  d8
r2  1.6798
r3  1.0928
a3  109.66
r4  1.0928
a4  109.66
d4  120.02
r5  1.0928
a5  109.66
d5  120.01
r6  1.0928
a6  109.66
r7  1.0928
a7  109.65
d7  120.03
r8  1.0928
a8  109.66
d8  120.02
tw  150.

$nbo print=0 $end
$DEL
  Lewis
$END
--Link1--
%mem=2gb
```

```
%nprocshared=8
%chk=rot_barr
#b3lyp/6-311++g** pop=nbo7del nosymm opt=z-matrix
CH3CH3 barrier, E(DEL), tau=160,-79.817911752 (0.01)
0 1
C
C   1   r2
H   1   r3   2  a3
H   1   r4   2  a4   3  d4
H   1   r5   2  a5   4  d5
H   2   r6   1  a6   3  tw
H   2   r7   1  a7   6  d7
H   2   r8   1  a8   7  d8
r2  1.6812
r3  1.0928
a3  109.70
r4  1.0928
a4  109.70
d4  120.02
r5  1.0928
a5  109.70
d5  120.01
r6  1.0928
a6  109.70
r7  1.0928
a7  109.69
d7  120.02
r8  1.0928
a8  109.70
d8  120.01
tw  160.

$nbo print=0 $end
$DEL
  Lewis
$END
--Link1--
%mem=2gb
%nprocshared=8
%chk=rot_barr
#b3lyp/6-311++g** pop=nbo7del nosymm opt=z-matrix
CH3CH3 barrier, E(DEL), tau=170,-79.817924097 (0.002)
0 1
C
C   1   r2
H   1   r3   2  a3
H   1   r4   2  a4   3  d4
H   1   r5   2  a5   4  d5
H   2   r6   1  a6   3  tw
H   2   r7   1  a7   6  d7
H   2   r8   1  a8   7  d8
r2  1.6826
r3  1.0928
a3  109.74
r4  1.0928
a4  109.74
d4  120.00
r5  1.0928
a5  109.74
d5  120.00
r6  1.0928
a6  109.74
r7  1.0928
a7  109.74
d7  119.99
r8  1.0928
a8  109.74
d8  120.00
tw 170.

$nbo print=0 $end
```

```
$DEL
  Lewis
$END
--Link1--
%mem=2gb
%nprocshared=8
%chk=rot_barr
#b3lyp/6-311++g** pop=nbo7del nosymm opt=z-matrix
CH3CH3 barrier, E(DEL), tau=180,-79.817928455 (0.00)
0 1
C
C   1   r2
H   1   r3   2  a3
H   1   r4   2  a4   3  d4
H   1   r5   2  a5   4  d5
H   2   r6   1  a6   3  tw
H   2   r7   1  a7   6  d7
H   2   r8   1  a8   7  d8
r2  1.6826
r3  1.0928
a3  109.74
r4  1.0928
a4  109.74
d4  120.00
r5  1.0928
a5  109.74
d5  120.00
r6  1.0928
a6  109.74
r7  1.0928
a7  109.74
d7  119.99
r8  1.0928
a8  109.74
d8  120.00
tw  180.

$nbo print=0 $end
$DEL
  Lewis
$END
--Link1--
--Link1--
%mem=2gb
%nprocshared=8
%chk=rot_barr
#b3lyp/6-311++g** pop=nbo7read nosymm opt=z-matrix freq
CH3NH2, E(FULL)=-95.8938909670 (0.00)
nu: 304,821,973,...
 Vibrational temperatures:    437.08  1180.77  1400.08  1518.14  1673.21
          (Kelvin)           1930.46  2100.26  2154.21  2182.47  2398.47
                             4261.82  4397.11  4448.64  5046.96  5158.75
 Zero-point correction=                          0.063793 (Hartree/Particle)
 Thermal correction to Energy=                   0.067231
 Thermal correction to Enthalpy=                 0.068175
 Thermal correction to Gibbs Free Energy=        0.040861
 Sum of electronic and zero-point Energies=           -95.830098
 Sum of electronic and thermal Energies=              -95.826660
 Sum of electronic and thermal Enthalpies=            -95.825716
 Sum of electronic and thermal Free Energies=         -95.853030
0 1
C
N   1   r2
H   1   r3   2  a3
H   1   r4   2  a4   3  d4
H   1   r5   2  a5   4  d5
H   2   r6   1  a6   3  tw
H   2   r7   1  a7   6  d7
r2  1.4656
r3  1.0927
a3  109.18
```

```
r4  1.0928
a4  109.17
d4  116.82
r5  1.1003
a5  115.17
d5  121.58
r6  1.0141
a6  111.18
tw  61.90
r7  1.0141
a7  111.18
d7  119.37

$nbo file=ch3nh2 $end
--Link1--
--Link1--
%mem=2gb
%nprocshared=8
%chk=rot_barr
#b3lyp/6-311++g** pop=nbo7del nosymm freq
CH3NH2, E(DEL)=-95.846654215 (0.00)
nu: 36,898,969,...
 Vibrational temperatures:     51.71  1292.39  1394.50  1517.79  1627.43
          (Kelvin)           1928.36  2094.39  2265.24  2275.13  2521.01
                             4585.94  4612.08  4793.03  5158.17  5251.56
 Zero-point correction=                         0.065503 (Hartree/Particle)
 Thermal correction to Energy=                  0.069373
 Thermal correction to Enthalpy=                0.070317
 Thermal correction to Gibbs Free Energy=       0.040963
 Sum of electronic and zero-point Energies=          -95.781151
 Sum of electronic and thermal Energies=             -95.777282
 Sum of electronic and thermal Enthalpies=           -95.776337
 Sum of electronic and thermal Free Energies=        -95.805691
0 1
C
N   1   r2
H   1   r3   2  a3
H   1   r4   2  a4   3  d4
H   1   r5   2  a5   4  d5
H   2   r6   1  a6   3  tw
H   2   r7   1  a7   6  d7
r2  1.6042
r3  1.0849
a3  107.90
r4  1.0857
a4  105.73
d4  119.78
r5  1.0848
a5  107.92
d5  119.80
r6  1.0111
a6  107.13
tw  2.16
r7  1.0111
a7  107.14
d7  115.17

$nbo print=0 $end
$DEL
  Lewis
$END
--Link1--
--Link1--
%mem=2gb
%nprocshared=8
%chk=rot_barr
#b3lyp/6-311++g**  nosymm opt=modredundant
CH3NH2 rot. barrier
 Summary of Optimized Potential Surface Scan (add -95.0 to energies):
                                 1                 2                 3                 4
5
```

Eigenvalues --	-0.890808 (1.93)	-0.891004 (1.81)	-0.891594 (1.44)	-0.892394 (0.94)	-0.893166 (0.45)
R1	1.471384	1.472949	1.472153	1.470745	1.468483
R2	1.093994	1.095362	1.096928	1.098369	1.099367
R3	1.094804	1.094692	1.094726	1.094316	1.093885
R4	1.094226	1.092759	1.091888	1.091465	1.091762
R5	1.011117	1.012136	1.013169	1.014000	1.014499
R6	1.011128	1.011990	1.013031	1.013936	1.014471
A1	111.770082	112.802905	113.730522	114.415772	114.857730
A2	110.683928	110.341061	110.004530	109.641919	109.418698
A3	111.901829	111.073137	110.344165	109.741145	109.386027
A4	107.581067	107.341744	107.148686	107.148760	107.351859
A5	107.105543	107.642559	108.118827	108.415452	108.403118
A6	107.574278	107.408973	107.240830	107.209553	107.137481
A7	112.223747	111.824287	111.466379	111.154770	111.033789
A8	112.226026	111.541097	110.964480	110.558014	110.546470
A9	107.828645	106.991515	106.351471	106.074983	106.197257
D1	-0.000157	-9.999916	-20.000124	-30.000380	-40.000506
D2	121.616998	109.769330	98.334943	87.530401	77.599131
D3	119.896748	110.046890	100.185842	90.386599	80.785680
D4	-118.486096	-130.183865	-141.479092	-152.082620	-161.614684
D5	-120.127145	-130.953111	-141.689417	-152.107252	-162.132411
D6	1.490010	-11.183865	-23.354351	-34.576471	-44.532774
D7	0.000157	9.999916	20.000124	30.000380	40.000506

	6	7	8	9	10
Eigenvalues --	-0.893707 (0.12)	-0.893891 (0.00)	-0.893687 (0.13)	-0.893151 (0.46)	-0.892414 (0.93)
R1	1.466938	1.465428	1.465078	1.464883	1.465910
R2	1.100122	1.100448	1.100293	1.099784	1.098703
R3	1.093306	1.092687	1.092143	1.091694	1.091615
R4	1.092096	1.092816	1.093424	1.094239	1.094764
R5	1.014466	1.014152	1.013326	1.012434	1.011462
R6	1.014459	1.014124	1.013383	1.012601	1.011780
A1	115.086098	115.187005	115.104682	114.882380	114.423369
A2	109.241572	109.177960	109.181162	109.349236	109.663360
A3	109.195400	109.198352	109.329546	109.590565	109.902642
A4	107.626276	107.944509	108.184914	108.305443	108.235916

A5	108.238009	107.907335	107.614940	107.312040	107.205221
A6	107.163512	107.127799	107.130735	107.109761	107.134695
A7	111.056677	111.203613	111.494522	111.827766	112.179201
A8	110.773057	111.191224	111.726613	112.260882	112.671957
A9	106.656234	107.192131	107.865212	108.362961	108.737306
D1	-50.000469	-60.000414	-70.000411	-80.000456	-90.000259
D2	68.328456	59.369083	50.786500	42.048798	33.109549
D3	71.166356	61.603206	51.862407	41.992800	31.818620
D4	-170.504720	-179.027297	172.649319	164.042053	154.928428
D5	-171.930647	178.428927	168.767856	159.119011	149.341801
D6	-53.601722	-62.201576	-70.445232	-78.831735	-87.548391
D7	50.000469	60.000414	70.000411	80.000456	90.000259

	11	12	13	14	15
Eigenvalues --	-0.891651 (1.41)	-0.891058 (1.78)	-0.890809 (1.93)	-0.890991 (1.82)	-0.891554 (1.47)
R1	1.467316	1.469268	1.471137	1.472217	1.471927
R2	1.097395	1.095611	1.094225	1.093069	1.092387
R3	1.091880	1.092646	1.093969	1.095639	1.097300
R4	1.095178	1.095252	1.094923	1.094382	1.093820
R5	1.010818	1.010533	1.011132	1.011984	1.013000
R6	1.011152	1.010734	1.011091	1.011821	1.012749
A1	113.767152	112.896786	111.943240	110.978041	110.145428
A2	110.194075	110.845511	111.766455	112.664644	113.449929
A3	110.255494	110.562561	110.690924	110.674527	110.532932
A4	107.984083	107.613388	107.136963	106.812104	106.659918
A5	107.168283	107.330711	107.528999	107.775940	107.957117
A6	107.213787	107.355478	107.546353	107.713017	107.874768
A7	112.413868	112.477432	112.217803	111.905849	111.568312
A8	112.857756	112.759728	112.228022	111.632971	111.041247
A9	108.777589	108.556867	107.810113	107.097111	106.503481
D1	-100.000291	-110.000399	-120.000353	-130.000123	-140.000167
D2	23.486398	13.166224	1.589743	-9.976460	-21.357322
D3	21.428133	10.816797	0.193225	-10.277524	-20.528492
D4	144.914823	133.983419	121.783322	109.746139	98.114353
D5	139.581885	129.747518	120.048527	110.385334	100.785836
D6	-96.931425	-107.085859	-118.361376	-129.591003	-140.571319

```
          D7                100.000291        110.000399        120.000353        130.000123
140.000167
                              16                17                18                19
     Eigenvalues --          -0.892328 (0.98)  -0.893093 (0.50)  -0.893655 (0.15)  -0.893888
(0.00)
          R1                  1.470670          1.468758          1.467005          1.465628
          R2                  1.092068          1.092131          1.092334          1.092726
          R3                  1.098731          1.099778          1.100299          1.100466
          R4                  1.093350          1.093000          1.092860          1.092687
          R5                  1.013886          1.014403          1.014507          1.014184
          R6                  1.013659          1.014241          1.014417          1.014158
          A1                109.472309        109.146024        109.020077        109.167405
          A2                114.107708        114.603689        114.942778        115.176129
          A3                110.303716        109.942527        109.586372        109.190574
          A4                106.739914        107.007084        107.439268        107.853002
          A5                107.999883        107.852374        107.573512        107.193403
          A6                107.993519        108.053913        108.022671        107.968801
          A7                111.260340        111.085889        111.081993        111.156425
          A8                110.609077        110.543151        110.701854        111.111314
          A9                106.177119        106.250643        106.607582        107.127364
          D1               -150.000410       -160.000388       -170.000418        179.999643
          D2                -32.244099        -42.304072        -51.761676        -60.796479
          D3                -30.468216        -40.032653        -49.340826        -58.531913
          D4                 87.288095         77.663663         68.897917         60.671966
          D5                 91.295003         81.888304         72.506846         63.105533
          D6               -150.948687       -160.415380       -169.254412       -177.690588
          D7                150.000410        160.000388        170.000418       -179.999643
0 1
C
N   1   r2
H   1   r3   2  a3
H   1   r4   2  a4   3  d4
H   1   r5   2  a5   4  d5
H   2   r6   1  a6   3  tw
H   2   r7   1  a7   6  d7
r2  1.4656
r3  1.0927
a3  109.18
r4  1.0928
a4  109.17
d4  116.82
r5  1.1003
a5  115.17
d5  121.58
r6  1.0141
a6  111.18
r7  1.0141
a7  111.18
d7  119.37
tw  0.
3 2 1 6 S 18 10.
--Link1--
--Link1--
%mem=2gb
%nprocshared=8
%chk=rot_barr
#b3lyp/6-311++g** pop=nbo7del nosymm opt=z-matrix
CH3NH2 (tau=0), E(DEL)= -95.846651723 (0.002)
0 1
C
N   1   r2
H   1   r3   2  a3
H   1   r4   2  a4   3  d4
H   1   r5   2  a5   4  d5
H   2   r6   1  a6   3  tw
H   2   r7   1  a7   6  d7
r2  1.6043
r3  1.0846
a3  108.01
r4  1.0857
a4  105.77
```

```
d4  119.77
r5  1.0851
a5  107.78
d5  119.80
r6  1.0111
a6  107.16
r7  1.0110
a7  107.14
d7  115.16
tw  0.

$nbo print=0 $end
$DEL
  Lewis
$END
--Link1--
%mem=2gb
%nprocshared=8
%chk=rot_barr
#b3lyp/6-311++g** pop=nbo7del nosymm opt=z-matrix
CH3NH2 (tau=10), E(DEL)=-95.846644356 (0.01)
0 1
C
N   1   r2
H   1   r3   2  a3
H   1   r4   2  a4   3  d4
H   1   r5   2  a5   4  d5
H   2   r6   1  a6   3  tw
H   2   r7   1  a7   6  d7
r2  1.6042
r3  1.0843
a3  108.15
r4  1.0855
a4  106.04
d4  120.01
r5  1.0857
a5  107.44
d5  119.71
r6  1.0109
a6  107.28
r7  1.0108
a7  107.16
d7  115.34
tw  10.

$nbo print=0 $end
$DEL
  Lewis
$END
--Link1--
%mem=2gb
%nprocshared=8
%chk=rot_barr
#b3lyp/6-311++g** pop=nbo7del nosymm opt=z-matrix
CH3NH2 (tau=20), E(DEL)= -95.846636374 (0.01)
0 1
C
N   1   r2
H   1   r3   2  a3
H   1   r4   2  a4   3  d4
H   1   r5   2  a5   4  d5
H   2   r6   1  a6   3  tw
H   2   r7   1  a7   6  d7
r2  1.6048
r3  1.0842
a3  108.16
r4  1.0853
a4  106.41
d4  120.16
r5  1.0862
a5  107.15
```

```
d5  119.73
r6  1.0106
a6  107.40
r7  1.0105
a7  107.25
d7  115.54
tw  20.

$nbo print=0 $end
$DEL
  Lewis
$END
--Link1--
%mem=2gb
%nprocshared=8
%chk=rot_barr
#b3lyp/6-311++g** pop=nbo7del nosymm opt=z-matrix
CH3NH2 (tau=30), E(DEL)=-95.846632640 (0.01)
0 1
C
N   1   r2
H   1   r3   2  a3
H   1   r4   2  a4   3  d4
H   1   r5   2  a5   4  d5
H   2   r6   1  a6   3  tw
H   2   r7   1  a7   6  d7
r2  1.6050
r3  1.0842
a3  108.07
r4  1.0849
a4  106.85
d4  120.30
r5  1.0867
a5  106.90
d5  119.76
r6  1.0102
a6  107.51
r7  1.0101
a7  107.36
d7  115.75
tw  30.

$nbo print=0 $end
$DEL
  Lewis
$END
--Link1--
%mem=2gb
%nprocshared=8
%chk=rot_barr
#b3lyp/6-311++g** pop=nbo7del nosymm opt=z-matrix
CH3NH2 (tau=40), E(DEL)=-95.84663238 (0.01)
0 1
C
N   1   r2
H   1   r3   2  a3
H   1   r4   2  a4   3  d4
H   1   r5   2  a5   4  d5
H   2   r6   1  a6   3  tw
H   2   r7   1  a7   6  d7
r2  1.6051
r3  1.0843
a3  107.89
r4  1.0846
a4  107.27
d4  120.40
r5  1.0871
a5  106.74
d5  119.80
r6  1.0099
a6  107.57
```

```
r7  1.0099
a7  107.46
d7  115.89
tw  40.

$nbo print=0 $end
$DEL
  Lewis
$END
--Link1--
%mem=2gb
%nprocshared=8
%chk=rot_barr
#b3lyp/6-311++g** pop=nbo7del nosymm opt=z-matrix
CH3NH2 (tau=50), E(DEL)= -95.846632763 (0.01)
0 1
C
N   1   r2
H   1   r3   2  a3
H   1   r4   2  a4   3  d4
H   1   r5   2  a5   4  d5
H   2   r6   1  a6   3  tw
H   2   r7   1  a7   6  d7
r2  1.6051
r3  1.0844
a3  107.63
r4  1.0844
a4  107.64
d4  120.45
r5  1.0872
a5  106.68
d5  119.85
r6  1.0098
a6  107.56
r7  1.0098
a7  107.54
d7  115.94
tw  50.

$nbo print=0 $end
$DEL
  Lewis
$END
--Link1--
%mem=2gb
%nprocshared=8
%chk=rot_barr
#b3lyp/6-311++g** pop=nbo7del nosymm opt=z-matrix
CH3NH2 (tau=60), E(DEL)=-95.846632611 (0.01)
0 1
C
N   1   r2
H   1   r3   2  a3
H   1   r4   2  a4   3  d4
H   1   r5   2  a5   4  d5
H   2   r6   1  a6   3  tw
H   2   r7   1  a7   6  d7
r2  1.6052
r3  1.0847
a3  107.30
r4  1.0842
a4  107.92
d4  120.44
r5  1.0872
a5  106.72
d5  119.91
r6  1.0098
a6  107.50
r7  1.0099
a7  107.56
d7  115.89
```

```
tw  60.

$nbo print=0 $end
$DEL
  Lewis
$END
--Link1--
%mem=2gb
%nprocshared=8
%chk=rot_barr
#b3lyp/6-311++g** pop=nbo7del nosymm opt=z-matrix
CH3NH2 (tau=70), E(DEL)=-95.846632500 (0.01)
0 1
C
N   1   r2
H   1   r3   2  a3
H   1   r4   2  a4   3  d4
H   1   r5   2  a5   4  d5
H   2   r6   1  a6   3  tw
H   2   r7   1  a7   6  d7
r2  1.6052
r3  1.0849
a3  106.92
r4  1.0841
a4  108.10
d4  120.38
r5  1.0869
a5  106.84
d5  119.98
r6  1.0101
a6  107.40
r7  1.0102
a7  107.51
d7  115.74
tw  70.

$nbo print=0 $end
$DEL
  Lewis
$END
--Link1--
%mem=2gb
%nprocshared=8
%chk=rot_barr
#b3lyp/6-311++g** pop=nbo7del nosymm opt=z-matrix
CH3NH2 (tau=80), E(DEL)=-95.846634570 (0.01)
0 1
C
N   1   r2
H   1   r3   2  a3
H   1   r4   2  a4   3  d4
H   1   r5   2  a5   4  d5
H   2   r6   1  a6   3  tw
H   2   r7   1  a7   6  d7
r2  1.6050
r3  1.0852
a3  106.53
r4  1.0841
a4  108.19
d4  120.28
r5  1.0865
a5  107.04
d5  120.07
r6  1.0104
a6  107.29
r7  1.0105
a7  107.41
d7  115.55
tw  80.

$nbo print=0 $end
```

```
$DEL
  Lewis
$END
--Link1--
%mem=2gb
%nprocshared=8
%chk=rot_barr
#b3lyp/6-311++g** pop=nbo7del nosymm opt=z-matrix
CH3NH2 (tau=90), E(DEL)=-95.846640184 (0.01)
0 1
C
N   1   r2
H   1   r3   2  a3
H   1   r4   2  a4   3  d4
H   1   r5   2  a5   4  d5
H   2   r6   1  a6   3  tw
H   2   r7   1  a7   6  d7
r2  1.6048
r3  1.0854
a3  106.18
r4  1.0842
a4  108.20
d4  120.17
r5  1.0860
a5  107.29
d5  120.17
r6  1.0107
a6  107.19
r7  1.0108
a7  107.29
d7  115.37
tw  90.

$nbo print=0 $end
$DEL
  Lewis
$END
--Link1--
%mem=2gb
%nprocshared=8
%chk=rot_barr
#b3lyp/6-311++g** pop=nbo7del nosymm opt=z-matrix
CH3NH2 (tau=100), E(DEL)=-95.846640184 (0.01)
0 1
C
N   1   r2
H   1   r3   2  a3
H   1   r4   2  a4   3  d4
H   1   r5   2  a5   4  d5
H   2   r6   1  a6   3  tw
H   2   r7   1  a7   6  d7
r2  1.6048
r3  1.0854
a3  106.18
r4  1.0842
a4  108.20
d4  120.17
r5  1.0860
a5  107.29
d5  120.17
r6  1.0107
a6  107.19
r7  1.0108
a7  107.29
d7  115.37
tw  100.

$nbo print=0 $end
$DEL
  Lewis
$END
```

```
--Link1--
%mem=2gb
%nprocshared=8
%chk=rot_barr
#b3lyp/6-311++g** pop=nbo7del nosymm opt=z-matrix
CH3NH2 (tau=110), E(DEL)=-95.846648413 (0.00)
0 1
C
N   1   r2
H   1   r3   2  a3
H   1   r4   2  a4   3  d4
H   1   r5   2  a5   4  d5
H   2   r6   1  a6   3  tw
H   2   r7   1  a7   6  d7
r2  1.6045
r3  1.0855
a3  105.91
r4  1.0844
a4  108.14
d4  120.06
r5  1.0855
a5  107.96
d5  120.25
r6  1.0110
a6  107.13
r7  1.0111
a7  107.19
d7  115.21
tw  110.

$nbo print=0 $end
$DEL
  Lewis
$END
--Link1--
%mem=2gb
%nprocshared=8
%chk=rot_barr
#b3lyp/6-311++g** pop=nbo7del nosymm opt=z-matrix
CH3NH2 (tau=120), E(DEL)=-95.846653476 (0.00)
0 1
C
N   1   r2
H   1   r3   2  a3
H   1   r4   2  a4   3  d4
H   1   r5   2  a5   4  d5
H   2   r6   1  a6   3  tw
H   2   r7   1  a7   6  d7
r2  1.6043
r3  1.0856
a3  105.77
r4  1.0848
a4  107.98
d4  119.96
r5  1.0851
a5  107.80
d5  120.28
r6  1.0111
a6  107.12
r7  1.0111
a7  107.12
d7  115.14
tw  120.

$nbo print=0 $end
$DEL
  Lewis
$END
--Link1--
%mem=2gb
%nprocshared=8
```

```
%chk=rot_barr
#b3lyp/6-311++g** pop=nbo7del nosymm opt=z-matrix
CH3NH2 (tau=130), E(DEL)=-95.846651417 (0.00)
0 1
C
N   1   r2
H   1   r3   2  a3
H   1   r4   2  a4   3  d4
H   1   r5   2  a5   4  d5
H   2   r6   1  a6   3  tw
H   2   r7   1  a7   6  d7
r2  1.6043
r3  1.0856
a3  105.82
r4  1.0852
a4  107.75
d4  119.88
r5  1.0847
a5  107.99
d5  120.25
r6  1.0111
a6  107.17
r7  1.0111
a7  107.11
d7  115.17
tw  130.

$nbo print=0 $end
$DEL
  Lewis
$END
--Link1--
%mem=2gb
%nprocshared=8
%chk=rot_barr
#b3lyp/6-311++g** pop=nbo7del nosymm opt=z-matrix
CH3NH2 (tau=150), E(DEL)=-95.846636303 (0.01)
0 1
C
N   1   r2
H   1   r3   2  a3
H   1   r4   2  a4   3  d4
H   1   r5   2  a5   4  d5
H   2   r6   1  a6   3  tw
H   2   r7   1  a7   6  d7
r2  1.6047
r3  1.0852
a3  106.38
r4  1.0863
a4  107.18
d4  119.78
r5  1.0842
a5  108.14
d5  120.04
r6  1.0106
a6  107.39
r7  1.0105
a7  107.24
d7  115.51
tw  150.

$nbo print=0 $end
$DEL
  Lewis
$END
--Link1--
%mem=2gb
%nprocshared=8
%chk=rot_barr
#b3lyp/6-311++g** pop=nbo7del nosymm opt=z-matrix
CH3NH2 (tau=160), E(DEL)=-95.846632843 (0.01)
```

```
0 1
C
N   1   r2
H   1   r3   2  a3
H   1   r4   2  a4   3  d4
H   1   r5   2  a5   4  d5
H   2   r6   1  a6   3  tw
H   2   r7   1  a7   6  d7
r2  1.6049
r3  1.0849
a3  106.78
r4  1.0868
a4  106.94
d4  119.74
r5  1.0841
a5  108.09
d5  119.92
r6  1.0103
a6  107.50
r7  1.0102
a7  107.35
d7  115.73
tw  160.

$nbo print=0 $end
$DEL
  Lewis
$END
--Link1--
%mem=2gb
%nprocshared=8
%chk=rot_barr
#b3lyp/6-311++g** pop=nbo7del nosymm opt=z-matrix
CH3NH2 (tau=170), E(DEL)=-95.846632652 (0.01)
0 1
C
N   1   r2
H   1   r3   2  a3
H   1   r4   2  a4   3  d4
H   1   r5   2  a5   4  d5
H   2   r6   1  a6   3  tw
H   2   r7   1  a7   6  d7
r2  1.6049
r3  1.0847
a3  107.19
r4  1.0872
a4  106.77
d4  119.72
r5  1.0842
a5  107.94
d5  119.83
r6  1.0100
a6  107.58
r7  1.099
a7  107.47
d7  115.91
tw  170.

$nbo print=0 $end
$DEL
  Lewis
$END
--Link1--
%mem=2gb
%nprocshared=8
%chk=rot_barr
#b3lyp/6-311++g** pop=nbo7del nosymm opt=z-matrix
CH3NH2 (tau=180), E(DEL)=-95.846633379 (0.01)
0 1
C
N   1   r2
```

```
H   1   r3   2  a3
H   1   r4   2  a4   3  d4
H   1   r5   2  a5   4  d5
H   2   r6   1  a6   3  tw
H   2   r7   1  a7   6  d7
r2  1.6047
r3  1.0844
a3  107.58
r4  1.0873
a4  106.68
d4  119.71
r5  1.0843
a5  107.73
d5  119.75
r6  1.0097
a6  107.61
r7  1.0097
a7  107.59
d7  116.08
tw  180.

$nbo print=0 $end
$DEL
  Lewis
$END
--Link1--
--Link1--
%mem=2gb
%nprocshared=8
%chk=rot_barr
#b3lyp/6-311++g** pop=nbo7read nosymm opt=z-matrix freq
HCONH2, E(full)=-169.954848506
nu:233,568,635,...
 Vibrational temperatures:    335.43   817.38   913.54  1492.61  1515.05
          (Kelvin)           1815.98  2038.15  2328.27  2577.26  4238.56
                             5148.90  5345.83
 Zero-point correction=                          0.045233 (Hartree/Particle)
 Thermal correction to Energy=                   0.048983
 Thermal correction to Enthalpy=                 0.049927
 Thermal correction to Gibbs Free Energy=        0.020701
 Sum of electronic and zero-point Energies=          -169.909615
 Sum of electronic and thermal Energies=             -169.905866
 Sum of electronic and thermal Enthalpies=           -169.904922
 Sum of electronic and thermal Free Energies=        -169.934148
0 1
H
C   1  r2
O   2  r3   1   a3
N   2  r4   1   a4   3  d4
H   4  r5   2   a5   3  d5
H   4  r6   2   a6   3  d6
r2   1.1059
r3   1.2115
a3   122.69
r4   1.3610
a4   112.38
d4   180.00
r5   1.0093
a5   119.42
d5   0.00
r6   1.0067
a6   121.42
d6   180.00
$nbo file=hconh2 $end
--Link1--
--Link1--
%mem=2gb
%nprocshared=8
%chk=rot_barr
#b3lyp/6-311++g** pop=nbo7del nosymm freq
HCONH2, E(DEL)=-169.769538009
```

```
nu: 197,500,835,...
 Vibrational temperatures:    282.81   719.63  1200.96  1427.56  1672.33
          (Kelvin)           1862.03  2054.75  2545.64  2635.60  5228.67
                             5239.23  5281.13
 Zero-point correction=                           0.047740 (Hartree/Particle)
 Thermal correction to Energy=                    0.051510
 Thermal correction to Enthalpy=                  0.052454
 Thermal correction to Gibbs Free Energy=         0.022881
 Sum of electronic and zero-point Energies=           -169.721798
 Sum of electronic and thermal Energies=              -169.718028
 Sum of electronic and thermal Enthalpies=            -169.717084
 Sum of electronic and thermal Free Energies=         -169.746657
0 1
H
C   1  r2
O   2  r3   1   a3
N   2  r4   1   a4   3  d4
H   4  r5   2   a5   3  d5
H   4  r6   2   a6   3  d6
r2   1.0478
r3   1.3037
a3   117.59
r4   1.5411
a4   128.20
d4   179.82
r5   1.0131
a5   106.08
d5   56.12
r6   1.0131
a6   106.16
d6   303.08
$nbo print=0 $end
$DEL
 Lewis
$END
--Link1--
--Link1--
%mem=2gb
%nprocshared=8
%chk=rot_barr
#b3lyp/6-311++g** nosymm pop=nbo7read opt=modredundant
HCONH2, E(full)=-169.954848506
 Summary of Optimized Potential Surface Scan (add -169.0 to energies):
                                1                 2                 3                 4
5
     Eigenvalues --           -0.954849 (0.00)  -0.954688 (0.10)  -0.954021 (0.52)  -0.952580
(1.42)  -0.950276 (2.87)
           R1                  1.105900          1.106040          1.106197          1.106500
1.106946
           R2                  1.211500          1.211373          1.210798          1.209402
1.208164
           R3                  1.361000          1.362785          1.367284          1.373444
1.379757
           R4                  1.009300          1.009728          1.010965          1.012046
1.013033
           R5                  1.006700          1.007359          1.008922          1.010554
1.012133
           A1                122.690000        122.649436        122.490422        122.369331
122.130860
           A2                112.380000        112.385256        112.519924        112.556113
112.669768
           A3                124.930000        124.941054        124.884813        124.840177
124.806521
           A4                119.420000        118.847886        117.638714        116.737063
116.064576
           A5                121.420000        120.529627        118.792788        116.812490
114.925973
           A6                119.160000        118.346865        116.511102        114.333219
112.149333
           D1                180.000000       -171.757085       -163.656455       -155.469287
-147.090356
```

D2	0.000000	-9.168882	-14.030585	-14.975499	-13.449826
D3	0.000000	9.999944	20.000035	29.999896	39.999919
D4	180.000000	172.588147	169.625905	170.493683	173.640449

	6	7	8	9	10
Eigenvalues --	-0.947171 (4.82)	-0.943417 (7.17)	-0.939236 (9.80)	-0.934900 (12.52)	-0.930735 (15.13)
R1	1.107214	1.107922	1.108087	1.108673	1.109073
R2	1.207001	1.205406	1.203846	1.202066	1.200156
R3	1.386917	1.394380	1.403201	1.411856	1.420985
R4	1.014063	1.014985	1.016149	1.017341	1.018596
R5	1.013638	1.015001	1.016562	1.017957	1.019410
A1	121.822284	121.516813	121.245516	120.984666	120.831305
A2	112.939107	113.237124	113.710003	114.287128	114.936617
A3	124.693460	124.580711	124.332968	124.051463	123.685324
A4	115.483566	115.035854	114.323541	113.469388	112.341871
A5	113.261434	111.692866	110.293645	109.135268	108.151445
A6	110.105639	108.195738	106.495485	105.068955	103.993102
D1	-138.367738	-129.262461	-119.594959	-109.376377	-98.439646
D2	-10.096850	-5.433397	0.363257	7.409102	15.756387
D3	49.999892	59.999949	69.999924	79.999925	89.999946
D4	178.270780	-176.170987	-170.041860	-163.214596	-155.804021

	11	12	13	14	15
Eigenvalues --	-0.927102 (17.41)	-0.924386 (19.12)	-0.923009 (19.98)	-0.948861 (3.76)	-0.951390 (2.17)
R1	1.109845	1.110187	1.110557	1.104925	1.105380
R2	1.198088	1.196485	1.195526	1.208549	1.209314
R3	1.429746	1.436280	1.439509	1.384760	1.377989
R4	1.019984	1.020775	1.020187	1.013360	1.011768
R5	1.020623	1.021369	1.020325	1.016016	1.014340
A1	120.739184	120.688562	120.626715	122.403678	122.503250
A2	115.585880	116.148056	116.471529	112.656334	112.551720
A3	123.324611	123.017909	122.893466	124.932969	124.944971
A4	110.934833	109.691033	109.052342	115.429402	116.666999
A5	107.526080	107.424846	108.500318	111.624592	113.475380
A6	103.403576	103.489385	105.016659	110.724868	112.812846
D1	-86.764170	-74.363629	-61.042867	-49.052516	-39.913640
D2	25.636826	37.474820	52.823699	-176.641794	-173.634263

```
          D3                99.999946       109.999913       119.999960       130.000032
139.999997
          D4              -147.599058      -138.161638      -126.133473         2.410754
6.279374
                             16               17               18               19
     Eigenvalues --          -0.953181 (1.05)  -0.954249 (0.38)  -0.954731 (0.07)  -0.954848
(0.00)
          R1                 1.105565         1.105835         1.106021         1.105965
          R2                 1.210302         1.210839         1.211562         1.211742
          R3                 1.371835         1.366665         1.362379         1.360805
          R4                 1.010004         1.008486         1.007103         1.006616
          R5                 1.012584         1.010993         1.009622         1.009141
          A1               122.599110       122.637282       122.658345       122.683609
          A2               112.547029       112.473229       112.409514       112.381861
          A3               124.850497       124.881378       124.926642       124.934530
          A4               118.071492       119.474162       120.746255       121.384993
          A5               115.291711       117.247329       118.767243       119.475501
          A6               114.985604       116.944192       118.569032       119.139360
          D1               -30.654510       -21.016047       -10.836557         0.004907
          D2              -172.048659      -172.238940      -174.854819      -179.854523
          D3               150.000061       160.000065       170.000061      -179.999974
          D4                 8.605911         8.777172         5.981799         0.140596
0 1
H
C   1  r2
O   2  r3   1   a3
N   2  r4   1   a4   3  d4
H   4  r5   2   a5   3  d5
H   4  r6   2   a6   3  d6
r2   1.1059
r3   1.2115
a3   122.69
r4   1.3610
a4   112.38
d4   180.00
r5   1.0093
a5   119.42
d5   0.00
r6   1.0067
a6   121.42
d6   180.00
3 2 4 5 S 18 10.
$nbo file=hconh2 archive $end
--Link1--
--Link1—
```

STERIC REPULSIONS

```
%mem=2gb
%nprocshared=8
%chk=he_he
#b3lyp/6-311++g** pop=nbo7read
He...He (R=5), -5.82708671068 (dE=0.00)
0 1
He
He   1   5.
$nbo file=he_he_5 archive steric $end
--Link1--
%mem=2gb
%nprocshared=8
%chk=he_he
#b3lyp/6-311++g** pop=nbo7read
He...He (R=4), -5.82707721566 (dE=0.007)
0 1
He
He   1   4.
$nbo file=he_he_4 archive steric $end
--Link1--
%mem=2gb
%nprocshared=8
%chk=he_he
```

```
#b3lyp/6-311++g** pop=nbo7read
He...He (R=3), -5.82701220107 (dE=0.05)
0 1
He
He   1   3.
$nbo file=he_he_3 archive steric $end
--Link1--
%mem=2gb
%nprocshared=8
%chk=he_he
#b3lyp/6-311++g** pop=nbo7read
He...He (R=2), -5.82522970796 (dE=1.17)
0 1
He
He   1   2.
$nbo file=he_he_2 archive steric $end
--Link1--
%mem=2gb
%nprocshared=8
%chk=he_he
#b3lyp/6-311++g** pop=nbo7read
He...He (R=1), -5.68164563835 (dE=91.27)
0 1
He
He   1   1.
$nbo file=he_he_1 archive steric $end
--Link1--
%mem=2gb
%nprocshared=8
%chk=chfchf_cis
#b3lyp/6-311++g** pop=nbo7read opt freq
cis-CHF=CHF (C2v), -277.142846492 (dE=0.00 kcal/mol)
nu: 236,510,771,...
 Vibrational temperatures:    340.26   733.63  1108.84  1115.38  1250.73
          (Kelvin)           1452.32  1617.68  1842.23  1987.87  2526.19
                             4626.36  4659.17
 Zero-point correction=                           0.036831 (Hartree/Particle)
 Thermal correction to Energy=                    0.040700
 Thermal correction to Enthalpy=                  0.041644
 Thermal correction to Gibbs Free Energy=         0.011166
 Sum of electronic and zero-point Energies=           -277.106015
 Sum of electronic and thermal Energies=              -277.102147
 Sum of electronic and thermal Enthalpies=            -277.101202
 Sum of electronic and thermal Free Energies=         -277.131681
0 1
C
C   1   1.3238
H   1   1.0810    2   122.96
F   1   1.3432    2   122.62  3   180.
H   2   1.0810    1   122.96  3     0.
F   2   1.3432    1   122.62  3   180.
$nbo file=chfchf_cis archive nrt plot steric $end
--Link1--
%mem=2gb
%nprocshared=8
%chk=chfchf_trans
#b3lyp/6-311++g** pop=nbo7read opt freq
trans-CHF=CHF (C2h), -277.141563885 (dE=0.80 kcal/mol)
nu: 317,334,551,...
 Vibrational temperatures:    456.51   480.63   793.18  1185.63  1300.77
          (Kelvin)           1628.85  1653.17  1851.01  1874.46  2504.93
                             4635.61  4646.00
 Zero-point correction=                           0.036435 (Hartree/Particle)
 Thermal correction to Energy=                    0.040427
 Thermal correction to Enthalpy=                  0.041371
 Thermal correction to Gibbs Free Energy=         0.010939
 Sum of electronic and zero-point Energies=           -277.105129
 Sum of electronic and thermal Energies=              -277.101137
 Sum of electronic and thermal Enthalpies=            -277.100193
 Sum of electronic and thermal Free Energies=         -277.130625
0 1
```

```
C
C   1   1.3234
H   1   1.0813    2   125.70
F   1   1.3499    2   120.01  3   180.
H   2   1.0813    1   125.70  3   180.
F   2   1.3499    1   120.01  3     0.
$nbo file=chfchf_trans archive nrt plot steric $end
--Link1--
%mem=2gb
%nprocshared=8
%chk=cis_2_butene_rb
#b3lyp/6-311++G** pop=nbo7read
cis_2_butene methyl rotation, E(tau=0)=-157.272643068 (dE=0.00)
Total steric exchange energy:   135.22 kcal/mol
dE(SXE)=2.01
dE(SXE;i,j)=2.06
0 1
               6          0.000000    1.590550   -0.521941
               6          0.000000    0.667983    0.663470
               6         -0.000000   -0.667983    0.663470
               1          0.000000    1.060210   -1.475268
               1         -0.878256    2.245715   -0.502836
               1          0.878256    2.245715   -0.502836
               1          0.000000    1.165182    1.631762
               1         -0.000000   -1.165182    1.631762
               6         -0.000000   -1.590550   -0.521941
               1         -0.000000   -1.060210   -1.475268
               1          0.878256   -2.245715   -0.502836
               1         -0.878256   -2.245715   -0.502836
$nbo file=cis_2_butene_0 archive steric nrt plot $end
--Link1--
%mem=2gb
%nprocshared=8
%chk=cis_2_butene_rb
#b3lyp/6-311++G** pop=nbo7read
cis_2_butene methyl rotation, E(tau=10)=-157.272543650 (dE=0.06)
Total steric exchange energy:   135.13 kcal/mol
dE(SXE)=1.92
dE(SXE;i,j)=2.01
0 1
               6          1.589478   -0.522182    0.000621
               6          0.667862    0.664514   -0.004684
               6         -0.668169    0.664048    0.000502
               1          1.068902   -1.466558   -0.164703
               1          2.350729   -0.422014   -0.780286
               1          2.126825   -0.595161    0.953478
               1          1.164573    1.632781    0.005117
               1         -1.165879    1.631916    0.005330
               6         -1.589091   -0.522555   -0.000207
               1         -1.057295   -1.474767   -0.020574
               1         -2.228099   -0.516279    0.890149
               1         -2.260238   -0.492870   -0.865899
$nbo file=cis_2_butene_10 archive steric nrt plot $end
--Link1--
%mem=2gb
%nprocshared=8
%chk=cis_2_butene_rb
#b3lyp/6-311++G** pop=nbo7read
cis_2_butene methyl rotation, E(tau=20)=-157.272268007 (dE=0.24)
Total steric exchange energy:   134.81 kcal/mol
dE(SXE)=1.60
dE(SXE;i,j)=1.87
0 1
               6          1.585613   -0.523088    0.001047
               6          0.667337    0.667895   -0.008226
               6         -0.668600    0.666167    0.000871
               1          1.093857   -1.440283   -0.327651
               1          2.440885   -0.354096   -0.660499
               1          1.993525   -0.698865    1.003585
               1          1.163762    1.635958    0.009250
               1         -1.168780    1.632661    0.009746
```

```
               6         -1.584069   -0.524527   -0.000485
               1         -1.046981   -1.473282   -0.039480
               1         -2.208401   -0.532613    0.900336
               1         -2.269550   -0.488162   -0.854525
$nbo file=cis_2_butene_20 archive steric nrt plot $end
--Link1--
%mem=2gb
%nprocshared=8
%chk=cis_2_butene_rb
#b3lyp/6-311++G** pop=nbo7read
cis_2_butene methyl rotation, E(tau=30)=-157.271883143 (dE=0.48)
Total steric exchange energy:   134.31 kcal/mol
dE(SXE)=1.10
dE(SXE;i,j)=1.65
0 1
               6          1.579485   -0.524684    0.001142
               6          0.666776    0.673153   -0.009334
               6         -0.669044    0.669391    0.000872
               1          1.136176   -1.395288   -0.486859
               1          2.513951   -0.300051   -0.520365
               1          1.848378   -0.813352    1.024027
               1          1.162982    1.640758    0.012225
               1         -1.173127    1.633808    0.010987
               6         -1.576331   -0.527371   -0.000709
               1         -1.031286   -1.471305   -0.049761
               1         -2.192169   -0.546216    0.905921
               1         -2.270223   -0.491288   -0.847994
$nbo file=cis_2_butene_30 archive steric nrt plot $end
--Link1--
%mem=2gb
%nprocshared=8
%chk=cis_2_butene_rb
#b3lyp/6-311++G** pop=nbo7read
cis_2_butene methyl rotation, E(tau=40)=-157.271489489 (dE=0.72)
Total steric exchange energy:   133.77 kcal/mol
dE(SXE)=0.56
dE(SXE;i,j)=1.32
0 1
               6          1.571893   -0.526597    0.000890
               6          0.665924    0.679087   -0.007938
               6         -0.669708    0.673593    0.000686
               1          1.199497   -1.332706   -0.636439
               1          2.568619   -0.262233   -0.360336
               1          1.694462   -0.931719    1.012000
               1          1.162707    1.646138    0.010767
               1         -1.178162    1.635501    0.010241
               6         -1.566859   -0.530810   -0.000814
               1         -1.011167   -1.468625   -0.050413
               1         -2.182764   -0.556492    0.905582
               1         -2.260693   -0.501505   -0.848344
$nbo file=cis_2_butene_40 archive steric nrt plot $end
--Link1--
%mem=2gb
%nprocshared=8
%chk=cis_2_butene_rb
#b3lyp/6-311++G** pop=nbo7read
cis_2_butene methyl rotation, E(tau=50)=-157.271200474 (dE=0.91)
Total steric exchange energy:   133.38 kcal/mol
dE(SXE)=0.17
dE(SXE;i,j)=0.94
0 1
               6          1.565698   -0.527991    0.000352
               6          0.665181    0.684119   -0.004359
               6         -0.670213    0.676916    0.000082
               1          1.286529   -1.252591   -0.770293
               1          2.602853   -0.241236   -0.184857
               1          1.541353   -1.049099    0.963838
               1          1.162322    1.650678    0.007353
               1         -1.182891    1.636592    0.005691
               6         -1.558634   -0.533606   -0.000513
               1         -0.994296   -1.467204   -0.033383
```

```
               1         -2.187877   -0.554624    0.896841
               1         -2.240189   -0.519146   -0.858559
$nbo file=cis_2_butene_50 archive steric nrt plot $end
--Link1--
%mem=2gb
%nprocshared=8
%chk=cis_2_butene_rb
#b3lyp/6-311++G** pop=nbo7read
cis_2_butene methyl rotation, E(tau=60)=-157.271092891 (dE=0.97)
Total steric exchange energy:   133.21 kcal/mol
dE(SXE)=0.00
dE(SXE;i,j)=0.56
0 1
               6          1.563242   -0.528484    0.000012
               6          0.664998    0.686084   -0.000089
               6         -0.670311    0.678434    0.000055
               1          1.398197   -1.159612   -0.879432
               1          2.614775   -0.235447   -0.002794
               1          1.402029   -1.156412    0.882483
               1          1.162543    1.652444   -0.000050
               1         -1.184448    1.637244    0.000183
               6         -1.555282   -0.534665   -0.000023
               1         -0.987068   -1.466519   -0.001014
               1         -2.210301   -0.540559    0.878703
               1         -2.211608   -0.539351   -0.877812
$nbo file=cis_2_butene_60 archive steric nrt plot $end
--Link1--
--Link1--
%mem=2gb
%nprocshared=8
%chk=cis_2_butene_rb
#b3lyp/6-311++G** opt=modredundant
cis_2_butene methyl rotation, E(tau)
0 1
C
C   1   r2
C   2   r3   1   a3
H   1   r4   2   a4   3  tau
H   1   r5   2   a5   4  tw5
H   1   r6   2   a6   4  tw6
H   2   r7   1   a7   3  d7
H   3   r8   2   a8   7  d8
C   3   r9   2   a9   7  d9
H   9   r10  3   a10  2  d10
H   9   r11  3   a11 10  d11
H   9   r12  3   a12 10  d12
r2  1.5
r3  1.3
a3  120.
r4  1.
a4  110.
tau 0.
r5  1.
a5  110.
tw5 120.
r6  1.
a6  110.
tw6 -120.
r7  1.
a7  120.
d7  180.
r8  1.
a8  120.
d8  0.
r9  1.5
a9  120.
d9  180.
r10 1.
a10 110.
d10 0.
r11 1.
```

```
a11 110.
d11 120.
r12 1.
a12 110.
d12 -120.
3 2 1 4  S  6  10.
$nbo file-propene steric nrt $end
--Link1--
--Link1--
```
